# Supplementary material for: Protocol for the systematic review of the reporting of transoral robotic surgery
Source: BMJ Open. 2018 Jan 23;8(1):e019198. doi: 10.1136/bmjopen-2017-019198 (PMC5786071; doi:10.1136/bmjopen-2017-019198)
Supplement: Supplementary file 1 [file bmjopen-2017-019198supp001.pdf]

**Appendix 1. Search strategy for OVID version of Medline**

1. Robotic Surgical Procedures/

2. ("robotic surgery" or "robotic surgeries" or "robot surgery" or "robotic surgical").mp.

3. 1 or 2

4. (transoral or trans-oral or "trans oral" or tors).mp.

5. exp Oropharyngeal Neoplasms/

6. ((oropharyngeal or oro-pharyngeal or tonsil* or "tongue base") and (cancer$ or neoplasm$ or carcinoma$ or tumo?r$)).mp.

7. 5 or 6

8. 3 and 4 and 7

9. Robotic Surgical Procedures/

10. ("robotic surgery" or "robotic surgeries" or "robot surgery" or "robotic surgical").mp.

11. 9 or 10

12. (transoral or trans-oral or "trans oral" or tors).mp.

13. exp Oropharyngeal Neoplasms/

14. ((oropharyngeal or oro-pharyngeal or tonsil* or "tongue base") and (cancer$ or neoplasm$ or carcinoma$ or tumo?r$)).mp.

15. 13 or 14

16. 11 and 12 and 15

17. Sleep Apnea, Obstructive/su [Surgery]

18. 17 and 3

19. 18 or 16
