# Supplementary material for: Protocol for the systematic review of the reporting of transoral robotic surgery
Source: BMJ Open. 2018 Jan 23;8(1):e019198. doi: 10.1136/bmjopen-2017-019198 (PMC5786071; doi:10.1136/bmjopen-2017-019198)
Supplement: Supplementary file 2 [file bmjopen-2017-019198supp002.pdf]

Systematic analysis of the reporting of surgical innovation: trans oral robotic surgery

Data Extraction Form

Section A: Citation details and funding arrangements. Complete for ALL PAPERS

A1: Unique citation ID: [ ] *(assigned by project lead)*

A2: 1st author: [ ]

A3: Journal: [ ]

A4: 2016 Impact Factor: [_ _._ _ _]

A5: Publication Year:

A6: Volume: [ ]

A7a: Page start: [ ]   A7b: Page end: [ ]

A8: Assessor: [ ]   (1=BM, 2=AG, 3=PR, 4=JMB, 5=NW, 6=other - add initials here __)

A9: Date of extraction: _ _/_ _/_ _ _ _

A10: Record any statement about conflict of interest (including declarations of no COI) including which author(s) it refers to and the precise nature of the COI here:

____

____

____

A11a: Does the paper include a statement about sponsorship and/or funding? **YES/NO**

A11b: If YES to A11a, was the funding provided by the manufacturer of the robot? **YES/NO**

A11c: If not funded by the robot manufacturer, what was the source of funding or sponsorship? __________

____

A12: Record the corresponding author's email address here: ______________________________________

END OF SECTION A

IF THE PAPER REPORTS A PRE-CLINICAL STUDY – GO TO SECTION B

IF THE PAPER REPORTS A CLINICAL STUDY – GO TO SECTION D

Section B: Preclinical studies (Complete for papers reporting animal and/or cadaveric studies)

(insert free text or select appropriate code to answer each question)

B1: Type of preclinical study ________ (1= animal stud, 2= cadaveric study, 3=combined)

B2: Country of study: _______ (1=UK, 2=North America, 3=EEA, 4=Multiple, 5=Other)

B3: Did the paper include a clear a priori hypothesis and/or present the rationale for the work? **Yes/No**

B3a: If YES, select the most appropriate category for that rationale: ________ (1=first preclinical study of TORS, 2=first preclinical study of a modification of TORS, 3=surgeon training, 4=testing for safety/efficacy/adverse events, 5=to gain FDA/NICE/Other approval, 6=other)

B3b If OTHER, record the statement here verbatim from the paper:

B4: Does the paper include a statement about the surgeons' previous experience of using TORS? **YES/NO**

B4a: If YES, record that statement here, verbatim from the paper:

B4**:** Does the discussion/conclusion present a clear rationale in favour of, or rejection of, progression to clinical studies? **Yes/No**

B6a: If yes, record the statement here verbatim from the paper:

END OF SECTION B

Section C: Critical appraisal of ANIMAL studies. Complete for all pre-clinical in vivo animal studies

For each of the items, indicate whether or not the criteria are fulfilled in the paper

	Item	Y/N/NA
C1	Does the TITLE accurately describe the content of the paper?	
C2	Does the ABSTRACT provide an accurate summary of the study including the species of animal used?	
C3	Does the BACKGROUND/INTRODUCTION include sufficient scientific information (incl. relevant references) to understand the motivation and context for the study, and explain the approach and rationale?	
C4	Are there clearly described primary and secondary OBJECTIVES, or statement of a specific HYPOTHESIS?	
C5	Is there a clear ETHICAL STATEMENT indicating the nature of the ethics permissions, relevant licenses, and national or institutional guidelines for the care and use of animals?	
C6	Is the STUDY DESIGN (METHODS) clearly described (e.g. number of groups, steps taken to minimise bias,)	
C7	Does the METHODS section clearly describe how the procedure was carried out?	
	C7a: DRUGS – anaesthesia and analgesia	
	C7b: SURGERY – how the procedure was carried out	
	C7c: What TIME of day it was carried out	
	C7d: WHERE it was carried out	
	C7e: Method of EUTHANASIA	
C8	Are there details of the ANIMALS used (species, strain, sex, age, weight). NB might be expressed as mean/median. Record species here: ________________________	
C9	Are there details of how the animals were HOUSED and CARED FOR (animal husbandry)	
C10	SAMPLE SIZE: Is a sample size calculation provided. Is the sample size justified by the authors?	
C11	ALLOCATION: are details and justification provided for how animals were allocated to a given intervention (or control)	
C12	Are the primary and secondary outcomes clearly defined?	
C13	STATISTICAL METHODS: Are these clearly described?	
C14	BASELINE DATA: For each group, are relevant characteristics reported at baseline? (health status, weight etc)	
C15	NUMBERS ANALYSED: Is there clear reporting of the number of animals in each group included in each analysis?	
C16	OUTCOMES: Are the results of each analysis reported?	
C17	ADVERSE EVENTS: Are all adverse events reported in each group?	
C17	MODIFICATIONS: Are any modifications to reduce adverse events/ improve outcomes described and justified?	
C18	DISCUSSION and INTERPRETATION: Does the discussion take into account the study objectives and hypotheses?	
C19	DISCUSSION: Do the authors reflect on the strengths and weaknesses of their work?	
C20	CONCLUSION: Do the authors comments on the generalisability/translation of their findings?	

END OF SECTION C

Section D: Clinical Studies

Section D: Clinical studies: study design and rationale

D1: Type of study:

Use this algorithm to help determine the most appropriate study design for this paper. Indicate the study design by marking the appropriate circle on the diagram. If you are unsure about the study design, please discuss with the senior author

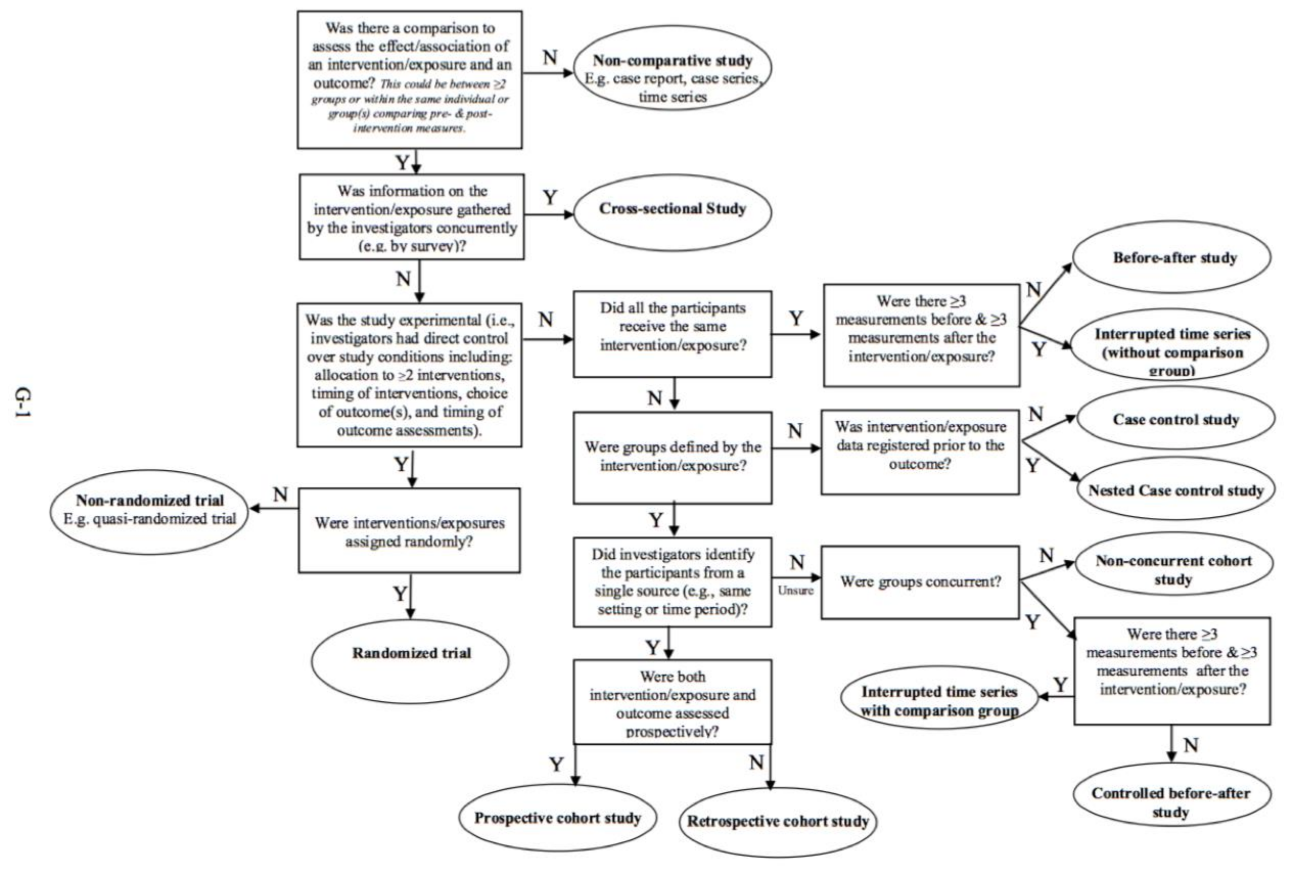

D2: Did the paper include a clear a priori hypothesis and/or present the rationale for the work? **Yes/No**

D2a: If YES, select the most appropriate category for that rationale: ________ (1=first preclinical study of TORS, 2=first preclinical study of a modification of TORS, 3=surgeon training, 4=testing for safety/efficacy/adverse events, 5=to gain FDA/NICE/Other approval, 6=other)

D2b If OTHER, record the statement here verbatim from the paper:

Section E: Data about the centre and surgeons (Complete for ALL papers reporting a CLINICAL study)

(insert free text or select appropriate code to answer each question)

E1: Country of study: _______ (1=UK, 2=North America, 3=EEA, 4=Multiple, 5=Other)

E2: Centre: _______ (1=single centre, 2=multi centre, NR=not reported)

E3: Number of centres if multiple: ______

E4: Type of centre: _______ (1=general/secondary care, 2=specialist/regional/supraregional/tertiary, 3=not specified)

E5: Is the caseload of the centre(s) reported? (e.g. number of cases normally treated per year) **YES/NO**

E5a: If YES, how many cases? _________

E6: How many surgeons were performing the intervention(s)? _______ (state number or 'NR' if not reported)

E7: Did the authors state any pre-specified criteria for surgeons to be eligible to perform the intervention?

YES/NO *(for example, does the paper state that participating surgeons must have performed it a given number of times?)*

E7a: If YES, what were these criteria and how (if at all) were they justified? (record here, verbatim from paper):

E8: Is there a generic statement about the expertise of those delivering the intervention(s)? **YES/NO** *(for example, does the paper state the number of cases participating surgeons have performed previously?)*

E8a: If YES, please record here verbatim from paper:

E9: If there a statement about how surgeon performance/expertise was monitored? YES/NO

E9a: If YES, please record the statement verbatim from paper here:

END OF SECTION E

Section F: Data about the patient participants (<u>Complete for ALL papers reporting a clinical study</u>)

(insert free text or select appropriate code to answer each question)

F1: Patients included in the study: ______ (1=adults only, 2= children only, 3=adults and children only, 4=not stated)

F2: Total number of participants: ______

F4: Average age of participants: _______ years (mean/median/mode – circle as appropriate)

F5: Sex of participants: _______ (%=male – calculate from paper if required)

F6: Did the authors include an a priori statement about inclusion and exclusion criteria for patients?

YES/NO

F6a: If yes, record in the table below:

**Inclusion criteria**	**Exclusion criteria**

F7: Were any of the criteria operator dependent (e.g. 'lack of expertise'). **YES/NO**

F7a: If YES, record here:____

F8: Did the authors include a statement about what happened to patients who did not meet the inclusion criteria to receive the intervention (or to participate if it was a comparative study)? **YES/NO**

F8a: If YES, please record the statement here, verbatim from the paper:

F9: Do the authors state that the study included consecutive patients? **YES/NO**

Section G: Data about governance and ethical factors (<u>Complete for ALL papers reporting a clinical study</u>)

(insert free text or select appropriate code to answer each question)

G1: Was there a statement confirming IRB*/ethics committee approval (**Yes/No**) (* Institutional Review Board)

G2: Was there a statement confirming individual patient consent? (**Yes/No**)

G2a: If yes, record this statement here (verbatim from paper) including any IRB/ethics approval number AND whether this consent was to the innovative procedure OR to the research project OR both:

G3: Was information provided about FDA/NICE/CE marking/clinical effectiveness approval? **YES/NO**

G3a: If YES, record the statement here, including whether that approval was for the device in general (e.g. robot) or for a specific clinical indication (e.g. oropharyngeal cancer):

G4: Was there a statement that patients were <u>specifically</u> informed about the innovative nature of the intervention? (**Yes/No**)

G4a: If yes, record this statement here (verbatim from paper):

G4b: Who gave this information to the patients (e.g. surgeon, nurse, other)? Please record any statement here: ____

G5: Did the paper report any amendment to the IRB/ethics approval AFTER the study had started? Include any statement here (including if the amendment was rejected, why the amendment was sought):

Section G continued: Data about governance and ethical factors (<u>Complete for ALL papers reporting a clinical study</u>)

G6: Was this study stated to be 'first-in-human' or 'first-in-man'? **YES/NO**

G6a: If YES, was there a statement confirming that the participant(s) were told this? **YES/NO**

G6b: If YES, record that statement here, verbatim from paper:

G7: Was information provided about the number of patients <u>declining</u> the intervention (**Yes/No**)

G7a: If yes, record this information here (verbatim from paper): (NB: this is different from the number of patients found to be ineligible for the intervention as recorded in section F).

G8: Did the authors describe additional sources of information about the intervention(s) provided for patients such as information leaflets, web pages or other sources? **YES/NO**

G8a: If YES, what were these sources:

G8b: If YES, were links or references to these sources included in the paper: **YES/NO**

G8c: If YES, have these sources been obtained? **YES/NO**

<u>If additional sources have been obtained, please retain for further analysis as part of the innovations repository project</u>

Section H: Data about the intervention (TORS) (Complete for ALL papers reporting a clinical study)

(insert free text or select appropriate code to answer each question)

H1: Does the study report at the OUTSET that it was of a modification/refinement/adaptation of TORS? **YES/NO**

H1a: If YES, what was the nature of the modification/refinement/adaptation? ________ (1=use of TORS for a new disease indication, 2=use of TORS in a new patient group, 3=modification of the robot (e.g. addition of an additional piece of equipment), 4=modification of operative technique (e.g. describing a new way for surgeons to operate the robot), 5=other)

H1b: Briefly describe the modification here: ____

____

H1c: If YES, was the rationale/justification for the modification described? **YES/NO**

H1d: If YES, briefly describe that rationale/justification here: ________________________________

____

H2: Does the study report any modifications/refinement/adaptation to TORS that took place AFTER the study had started?

H2a: If YES, what was the nature of the modification/refinement/adaptation? ________ (1=use of TORS for a new disease indication, 2=use of TORS in a new patient group, 3=modification of the robot (e.g. addition of an additional piece of equipment), 4=modification of operative technique (e.g. describing a new way for surgeons to operate the robot), 5=other)

H2b: Briefly describe the modification here: ____

____

H2c: If YES, was the rationale/justification for the modification described? **YES/NO**

H2d: If YES, briefly describe that rationale/justification here: ________________________________

____

H3: If there WAS a modification, is it reported that patients were told about this? **YES/NO**

H4: If YES, record the statement here, verbatim from paper (including any detail about how this information was given – e.g. leaflets):

END OF SECTION H

Section I: Data about the intervention (contd)) (Complete for ALL papers reporting a clinical study)

This section considers the component parts of the intervention based on Blencowe's typology. Complete the table

	Component	Described in paper? Y/N	If YES, verbatim description from the paper:
I1	Device (TORS) set-up/settings/positioning (not positioning of patient)		
I2	'Pre-incision' (skin preparation, draping, positioning of patient)		
I3	Incision		
I4	Access		
I5	Dissection		
I6	Resection		
I7	Haemostasis		
I8	Insertion of adjuncts (e.g. drains)*		
I9	Closure		

* Do not include tracheostomy here. If tracheostomy is described, please record this in the co-interventions section (section L).

Section J: Data about the <u>comparator</u> interventions <u>(Complete for ALL papers reporting a COMPARATIVE CLINICAL study)</u>

(insert free text or select appropriate code to answer each question) IF THERE IS MORE THAN ONE COMPARATOR (E.G. A THREE-GROUP TRIAL) PLEASE PRINT EXTRA PAGES AS REQUIRED

J1: Which category best describes the comparator: _____ (1= a different transoral surgical technique (e.g. transoral micro laser), 2= other surgery (e.g. lip split and mandibulotomy), 3=radiotherapy, 4= chemotherapy, 5=chemoradiotherapy, 6=other)

J1a: If 'other' please describe here: ____

J2: Does the paper describe an a priori hypothesis/rationale for selecting this comparator? **YES/NO**

J2a: If YES, please record the statement verbatim here:

J3: Does the study report at the <u>OUTSET</u> how patients were selected to be in either the comparator or intervention group(s) **YES/NO** (NB: not applicable if the paper is reporting a RCT)

J3a: If YES, please record the statement here, verbatim from the paper:

END OF SECTION J

Section K: Data about the comparator interventions (contd) (Complete for ALL papers reporting a COMPARATIVE CLINICAL study where the comparator is SURGICAL)

This section considers the component parts of the comparator intervention based on Blencowe's typology. Complete the table if the comparator is surgical. Do not complete if the comparator is non-surgical. PRINT AND COMPLETE MULTIPLE COPIES IF THERE WAS >1 COMPARATOR

	**Component**	**Described in paper? Y/N**	**If YES, verbatim description from the paper:**
K1	Device (e.g. laser or other instrument) set-up/settings/positioning (not positioning of patient)		
K2	'Pre-incision' (skin preparation, draping, positioning of patient)		
K3	Incision		
K4	Access		
K5	Dissection		
K6	Resection		
K7	Haemostasis		
K8	Insertion of adjuncts (e.g. drains)*		
K9	Closure		

* Do not include tracheostomy here. If tracheostomy is described, please record this in the co-interventions section (section L).

Section L: Data about CO-INTERVENTIONS (<u>Complete for ALL papers reporting a CLINICAL study</u>)

Complete the table for each of the co-interventions listed. The INTERVENTION refers to TORS, the comparator to an operative (surgical) comparator group where appropriate. If there was >1 comparator, please print additional copies of the table as required.

	**Co-intervention**	**Description provided in the paper (Y/N)**	**If YES: Verbatim description of the co-intervention (please include any variance in how the co-intervention was described between intervention and comparator group(s)**
L1	Pre-operative imaging		
L2	Pre-operative antibiotics		
L3	Pre-operative analgesia		
L4	Pre-operative fluids		
L5	Pre-operative lines or tubes		
L6	Other pre-op medication		
L7	Thromboprophylaxis		
L8	Other (state)		
L9	Anaesthesia		
L10	Peri-op analgesia		
L11	Peri-op fluids		
L12	Peri-op antibiotics		
L13	Tracheostomy		
L14	Other peri-op lines or tubes		
L15	Peri-op monitoring (pulse oximetry, invasive arterial, temperature)		
L16	Peri-op imaging		
L17	Peri-op thromboprophylaxis		
L18	Other (state)		
L19	Post-op fluids		
L20	Post-op analgesia		
L21	Post-op Antibiotics		
L22	Post-op lines and tubes		
L23	Post-op tracheostomy care		
L24	Intensive care		
L25	Physiotherapy		
L26	Speech and language therapy		
L27	Dietitian therapy		
L28	Other		

Section L: Data about CO-INTERVENTIONS (contd) (Complete for ALL papers reporting a CLINICAL study)

(insert free text or select appropriate code to answer each question)

L29: Does the study provide a statement about team expertise in delivering the co-intervention(s)? (e.g. anaesthetic team expertise in TORS, operating theatre support staff training or expertise) **YES/NO**

L29a: If YES, please record the statement here verbatim from the paper:

L30: Was there any reported modification to any co-intervention AFTER the study had started? YES/NO

L30a: If YES, describe that modification here:

L31: If this was a comparative study, was the modification in the co-intervention the SAME in both groups? YES/NO

L31A: If NO, how did the modification differ between groups?

L31b: If the co-intervention was different between groups, did the authors provide any rationale for that modification? **YES/NO**

L31c: If YES, please record that statement here verbatim from paper:

Section M: Outcomes reported (Complete ALL papers reporting a clinical study)

Use this table of outcome domain codes and their descriptors to help complete the next three sections

Domain code	Outcome domain	Definition
Outcome theme: adverse events		
1	Surgical complications	Any short or long term deviation from the normal post-operative course including symptomatic and asymptomatic complications but excluding death
2	Peri-operative technical outcomes	Outcomes recorded directly in the operating theatre, anaesthesia, pre-surgery and immediate recovery before return to the ward/intensive care – e.g. length of procedure, blood loss, or any technical aspects of the procedure
3	Mortality (<30 days)	Outcomes related to short-term survival/death rates and cause of death
4	Mortality (> 30 days)	Outcomes related to long-term survival/death rates and cause of death
Outcome theme: clinical effectiveness		
5	Overall survival	An objective measure of overall survival (at a given time) regardless of cause of death
6	Surrogate survival outcomes	Outcomes relating to survival but which are not overall survival – e.g. disease-free survival, progression-free survival, disease-specific survival
7	Tumour clearance	Outcomes related to achieving tumour clearance (e.g. clear margins)
8	Recurrence of disease	Outcomes related to the recurrence of disease – e.g. cancer or sleep apnoea. Recurrence may be local, regional or distant
9	Treatment pathway outcomes	Outcomes related to the flow of patients through the healthcare system – e.g. length of hospital stay, readmission rates
Outcome theme: physical symptoms, signs and measurements		
10	Haematological or biochemical markers	Outcomes related to the reporting of blood, tissue or biochemical markers – e.g. haemoglobin
11	Physiological measurements	Outcomes related to physiological measurements of function – e.g. sleep studies in treatment of obstructive sleep apnoea
12	Observer assessment of symptoms and function	Signs and symptoms of cancer or sleep apnoea reported to or by clinicians or health care providers directly, and those assessed in clinical practice
Outcome theme: issues relating to quality of life and wellbeing after surgery		
13	Patient-reported outcomes	Qualitative factors, generally assessed by questionnaires, reflecting aspects of a patient's daily life including activities, employment, eating behaviours, psychological health, emotional health, sexual life, sleep function
Outcome theme: health economics		
14	Cost effectiveness	Outcomes related to health economics including cost-effectiveness analyses/modelling, QALYs.
Outcome theme: other/additional/new outcomes		
15	Other	Any outcomes that cannot be categorised into one of the domains listed above

Section N: Outcomes reported. Please complete for all outcomes reported (you may need to check methods and results sections for details) *Print multiple copies of this page if needed. NB: if any PATIENT-REPORTED OUTOMES, please also complete sections O +/- P*

Outcome name	Domain code	Where reported in paper (1=methods, 2=results, 3=discussion, 4=tables/figures)	Is outcome defined (Y/N)	Verbatim definition	Any citation for a definition (Y/N)	Who ASSESSED the outcome ('NR' if not reported)	Primary or secondary outcome? ('NR' if not reported)

Section N (contd): Outcomes reported. Please complete for all outcomes reported (you may need to check methods and results sections for details) *Print multiple copies of this page if needed. NB: if any PATIENT-REPORTED OUTOMES, please also complete sections O +/- P*

Outcome name	Domain code	Where reported in paper (1=methods, 2=results, 3=discussion, 4=tables/figures)	Is outcome defined (Y/N)	Verbatim definition	Any citation for a definition (Y/N)	Who ASSESSED the outcome ('NR' if not reported)	Primary or secondary outcome? ('NR' if not reported)

Multiple event – print and insert further copies as required

Section O: Patient reported outcomes – *see Guidance notes for a definition*

O: Complete these questions for any patient reported outcome (one page for each PRO)

O1: Patient reported outcome measure:

i.e. Record verbatim the instrument used to measure

the PRO (e.g. QLQ-C30)

O2: If the study was an RCT, was this PRO the 1° outcome? _________ *(1=YES, 2=NO, 3=Not applicable, 4=UNSURE)*

O3: Is this PRO included on the e-provide database (https://eprovide.mapi-trust.org): **YES/NO**

If study is an RCT please complete sections J and K. If not, complete section J only

The following 12 questions are based on the Efficace criteria. See explanatory notes for guidance on completing this section. Tick appropriate box to answer each question.

O3: *A priori* hypothesis stated?

1=DOMAIN	2=DIRECTION	3=BOTH	4=NONE	5=N/A

	1=YES	2=NO	3=N/A
O4: Rationale for instrument reported?			
O5: Psychometric properties reported?			
O6: Cultural validity verified?			
O7: Adequate domains covered?			
O8: Instrument administration reported?			
O9: Baseline compliance reported?			
O10: Timings of assessments documented?			
O11: Missing data documented?			
O12: Clinical significance addressed?			
O13: Presentation of results in general?			

O14: Standard of HRQL reporting:

1=High	2=Low	3=N/A

END OF SECTION O

Multiple event – print and insert further copies as required

Section P: Patient reported outcomes – complete this section ONLY IF RANDOMISED CONTROLLED TRIAL

Section P: CONSORT Patient Reported Outcomes Extension

If the paper is an RCT and includes PROs, please also complete this CONSORT-PRO extension assessment: (http://jama.jamanetwork.com/article.aspx?articleid=1656259) See explanatory notes for further guidance. Tick appropriate box.

	**CONSORT-PRO extension checklist item**	**1 Yes**	**2 No**	**3 Unsure**
P1	PRO should be identified in abstract as a primary or secondary outcome			
P2	The PRO hypothesis should be stated and relevant domains identified, if applicable			
P3	Evidence of PRO instrument validity should be provided or cited if available, including the person completing the PRO and methods of data collection			
P4	Statistical approaches for dealing with missing data are explicitly stated			
P5	PRO-specific limitations and implications for generalisability and clinical practice are stated			

If 'UNSURE' for any, put paper forward for discussion with senior author.

END OF SECTION P

Section Q: Critical appraisal: CONSORT checklist for RANDOMISED TRIALS (*tick appropriate box in answer to each checklist item)*

Section/topic		Checklist item	1 YES	2 NO	3 N/A	4 UNSURE
**Title/abstract**	Q1	Identified as RCT in title				
	Q2	Structured abstract of trial design, methods, results, conclusions				
**Introduction**	Q3	Scientific background and explanation of rationale				
	Q4	Specific objectives and hypotheses				
**Methods**	Q5	Description of trial design including allocation ratio				
	Q6	Important changes to methods after trial commencement (e.g. eligibility criteria) explained				
**Eligibility**	Q7	Eligibility criteria for participants described				
**Settings**	Q8	Settings and locations of data collection described				
**Interventions**	Q9	Interventions for each group with sufficient detail to allow replication described				
**Outcomes**	Q10	Pre-specified primary and secondary outcome measures completely defined, including how and when they were assessed				
	Q11	Any changes to trial outcomes after trial commencement described, with reasons				
**Sample Size**	Q12	Explanation of how sample size was determined				
	Q13	If applicable, explanation of any interim analyses and stopping guidelines				
**Randomisation**	Q14	Description of method used to generate random allocation sequence				
	Q15	Description of type of randomisation				
	Q16	Description of mechanism used to implement random allocation sequence (e.g. sequentially numbered containers) including steps taken to conceal the sequence until interventions were assigned				
	Q17	Description of who generated the random allocation sequence, who enrolled participants, and who assigned participants to interventions				
**Blinding**	Q18	Description of any blinding if applicable				
**Statistics**	Q19	Description of statistical methods used to compare groups for primary and secondary outcomes				
		Description used for additional analyses (e.g. subgroup analyses, adjusted analyses)				
**Results**	Q20	Description of participant flow (in each group, number of participants randomly assigned, who received intended treatment, analysed for each primary outcome)				
	Q21	For each group, description of losses and exclusions after randomisation, with reasons				
	Q22	Dates defining the periods of recruitment and follow-up				
	Q23	Description of why the trial ended or was stopped				
**Baseline data**	Q24	Includes a table showing baseline demographics and clinical characteristics for each group				
	Q25	Details of number of participants (denominator) from each group included in each analysis and whether analysis was by original assigned group				
**Outcomes and estimation**	Q26	Results and an estimation of effect size and its precision (e.g. 95% CI) for each primary and secondary outcome for each group				
	Q27	Where applicable, for binary outcomes, inclusion of both absolute and relative effect sizes (*NB only a recommendation rather than requirement in CONSORT*)				
	Q28	Reporting of results of any other analyses performed (e.g. subgroup analyses)				
**Harms**	Q29	Reporting of all important harms or unintended effects in each group				
**Discussion**	Q30	Description of trial limitations, addressing of sources of potential bias, imprecision				
**Generalisability**	Q31	Discussion of generalisability (external validity, applicability) of trial results				
**Interpretation**	Q32	Interpretation that is consistent with results, and that balances benefits and harms and considers other relevant evidence				
**Other information**	Q33	Registration number and name of trial registry included				
	Q34	Details of where to access the full trial protocol, if available				
	Q35	Statement of sources of funding and other support (e.g. supply of drugs) and the role of any funders				

Refer to guidance notes for full explanation of terms

END OF SECTION Q

Section R: Cochrane tool for assessment of risk of bias for RANDOMISED TRIALS

See explanatory notes for guidance on completing, and/or the Cochrane website:

Tick appropriate box to answer each question

(http://ohg.cochrane.org/sites/ohg.cochrane.org/files/uploads/Risk%20of%20bias%20assessment%20tool.pdf)

	**Judgement item**	**1 YES**	**2 NO**	**3 UNSURE**
R1	Was the allocation sequence adequately generated?			
R2	Was the allocation adequately concealed?			
R3	Was knowledge of the allocated intervention adequately prevented during the study? (i.e. was there adequate blinding?)			
R4	Were incomplete outcome data adequately addressed?			
R5	Are reports of the study free of suggestion of selective outcome reporting?			
R6	Was the study apparently free of other problems that could it at a high risk of bias?			
R7	If NO, please specify other sources of bias: *(free text)*			
R8:	What is the overall risk of bias? *(use descriptor and code key to apply overall risk level)* **CODE KEY: 1=LOW, 2=MODERATE, 3=HIGH**	**If any 'UNSURE' discuss with senior author**		

**Risk level**	Description	**Code**
**LOW**	More than 4 'YES' responses	
**MODERATE**	3-4 'YES' responses	
**HIGH**	Fewer than 3 'YES' responses	

R9: Any other comments on bias? *(free text)*

END OF SECTION R

Section S: Critical appraisal: Complete this if paper is a CASE STUDY/CASE REPORT

Select most appropriate response for each statement

	Yes	No	Unclear	Not applicable
S1: Were the patient's demographic characteristics clearly described?				
S2: was the patient's history clearly described and presented as a timeline				
S3: Was the current clinical condition of the patient on presentation clearly described?				
S4: Were diagnostic tests or assessment methods and the results clearly described?				
S5: Was the intervention(s) or treatment procedure(s) clearly described?				
S6: Was the post-intervention clinical condition clearly described?				
S7: Were adverse events (harms) or unanticipated events identified and described?				
S8: Does the case report provide takeaway lessons?				

END OF SECTION S

Section T: Critical appraisal: Complete this if paper is a CASE SERIES

Select most appropriate response for each statement

	Yes	No	Unclear	Not applicable
T1: Were there clear criteria for inclusion in the case series?				
T2: Was the condition measured in a standard, reliable way for all participants included in the case series?				
T3: Were valid methods used for identification of the condition of all participants included in the case series?				
T4: Did the case series have consecutive inclusion of participants?				
T5: Did the case series have complete inclusion of participants?				
T6: Was there clear reporting of the demographics of the participants in the study?				
T7: Was there clear reporting of clinical information of the participants?				
T8: Were the outcomes or follow-up results of cases clearly reported?				
T9: Was there clear reporting of the presenting site(s)/clinic(s) demographic information?				
T10: Was statistical analysis appropriate?				

END OF SECTION T

Section U: Critical Appraisal: Complete this if paper is a COHORT STUDY

Select most appropriate response for each statement

	Yes	No	Unclear	Not applicable
U1: Were the groups similar and recruited from the same population?				
U2: Were the exposures measured similarly to assign people to both exposed and unexposed groups?				
U3: Was the exposure measured in a valid and reliable way?				
U4: Were the confounding factors identified?				
U5: Were strategies to deal with confounding factors stated?				
U6: Were the groups/participants free of the outcome at the start of the study (or at the moment of exposure?)				
U7: Were the outcomes measured in a valid and reliable way?				
U8: Was the follow up time reported and sufficient to be long enough for outcomes to occur?				
U9: Was follow-up complete, and if not, were the reasons to loss to follow-up described and explored?				
U10: Were strategies to address incomplete follow-up utilised?				
U11: Was appropriate statistical analysis used?				

END OF SECTION U

Section V: Critical Appraisal: Complete this if paper is a CROSS-SECTIONAL STUDY

Select most appropriate response for each statement

	Yes	No	Unclear	Not applicable
V1: Were the criteria for inclusion in the sample clearly defined?				
V2: Were the study subjects and the setting described in detail?				
V3: Was the exposure measured in a valid and reliable way?				
V4: Were objective, standard criteria used for measurement of the condition?				
V5: were confounding factors identified?				
V6: Were strategies to deal with confounding factors stated?				
V7: Were the outcomes measured in a valid and reliable way?				
V8: Was appropriate statistical analysis used?				

END OF SECTION V

Section W: Critical Appraisal. Complete this if paper is a NON-RANDOMISED COMPARATIVE STUDY ('QUASI-EXPERIMENTAL)

Select most appropriate response for each statement

	Yes	No	Unclear	Not applicable
W1: Is it clear in the study what is the 'cause' and what is the 'effect' (i.e. there is no confusion about which variable comes first)?				
W2: Were the participants included in any comparisons similar?				
W3: Were the participants included in any comparisons receiving similar treatment/care, other than the exposure or intervention of interest?				
W4: Was there a control group?				
W5: Were there multiple measurements of the outcome both pre- and post- the intervention/exposure?				
W6: Was follow-up complete, and if not, was follow-up adequately reported and strategies to deal with loss to follow-up employed?				
W7: Were the outcomes of participants included in any comparisons measured in the same way?				
W8: Were outcomes measured in a reliable way?				
W9: Were appropriate statistical analyses used?				

END OF SECTION W

Section X: Additional source(s) of information?

X1: Is any additional source required (e.g. from a study protocol)? **Yes/No**

X1a: Has the additional source of data been found? **Yes/no**

X1b: If yes, what source(s)?

X1c: What additional data did this source(s) add that was not found in the full paper?

X1d: has this additional data been entered at the appropriate place on this form? **YES/NO**

X2: Did this paper cite any references to earlier work? **YES/NO**

X2a: If YES, have these additional papers been obtained? **YES/NO**

X2b: If No, why not? (e.g. unable to obtain full paper) ____

Any additional papers obtained should have a unique citation ID assigned and full data extraction completed

Any additional sources (PILS, protocols etc) should be saved as PDF and sent to sian.cousins@bristol.ac.uk **for distribution to the appropriate researchers as part of the INFORM project)**

END OF SECTION X

Section Y: Summing up: Considering the DISCUSSION and CONCLUSION of the paper

Please read and re-read the discussion and/or conclusion *section of the paper before completing this section. Note: some of the information applicable to this section may also be found in the introduction*

X1:Select one of the following statements to most closely reflect the authors' conclusion about TORS from the findings of their study

	**Statement**	**Tick here if most appropriate**
Y1a	The authors recommend further INNOVATION (or development/refinement/adaptation) of TORS is required	
Y1b	The authors recommend further EVALUATION of TORS is required (e.g. they recommend evaluation in an RCT)	
Y1c	The authors recommend ADOPTION of TORS into routine clinical practice	
Y1d	The authors recommend STOPPING the use of TORS in routine clinical practice	
Y1e	The authors make a different conclusion to those above. If so, record their conclusion here:	

The following items relate to implementation and how the authors describe it. Please tick the most appropriate box to respond to each question in the table:

	Tick if applies
**Did the authors consider potential challenges in implementing the intervention more widely?**	
Training others/learning curve/workload or volume limiting patient pool	
Cost	
Competing interventions	
Lack of perceived need for a new intervention	
Resistance to change	
Other. Please state here:	
**Did the authors consider the role of any of the following elements in the generalisation of the intervention to other settings?**	
Specific surgeon(s)	
Specific team(s)	
Perioperative care	
Health care system	
Patient population	
Other. Please state here:	
**Did the authors describe any strategies for promoting engagement and change?**	
Raising profile through publication/presentation at conferences	
Workshops	
Formalised credentialing programme	
Engagement with national specialty/disease groups	
Engagement with national regulatory bodies	
Engagement with patient groups	
Other. Please state here:	

Section Z: Summing up: Categorisation of the study using the IDEAL framework. (Please read the guidance notes to help you complete this section)

Z1: Did the authors state an IDEAL stage? YES/NO. If YES, what was that stage? ________________________

Z2: Use the flow diagram below to classify the study by IDEAL stage. Indicate your selection by circling the appropriate box If it is not possible to classify the study, please state the reason here:____________________

____

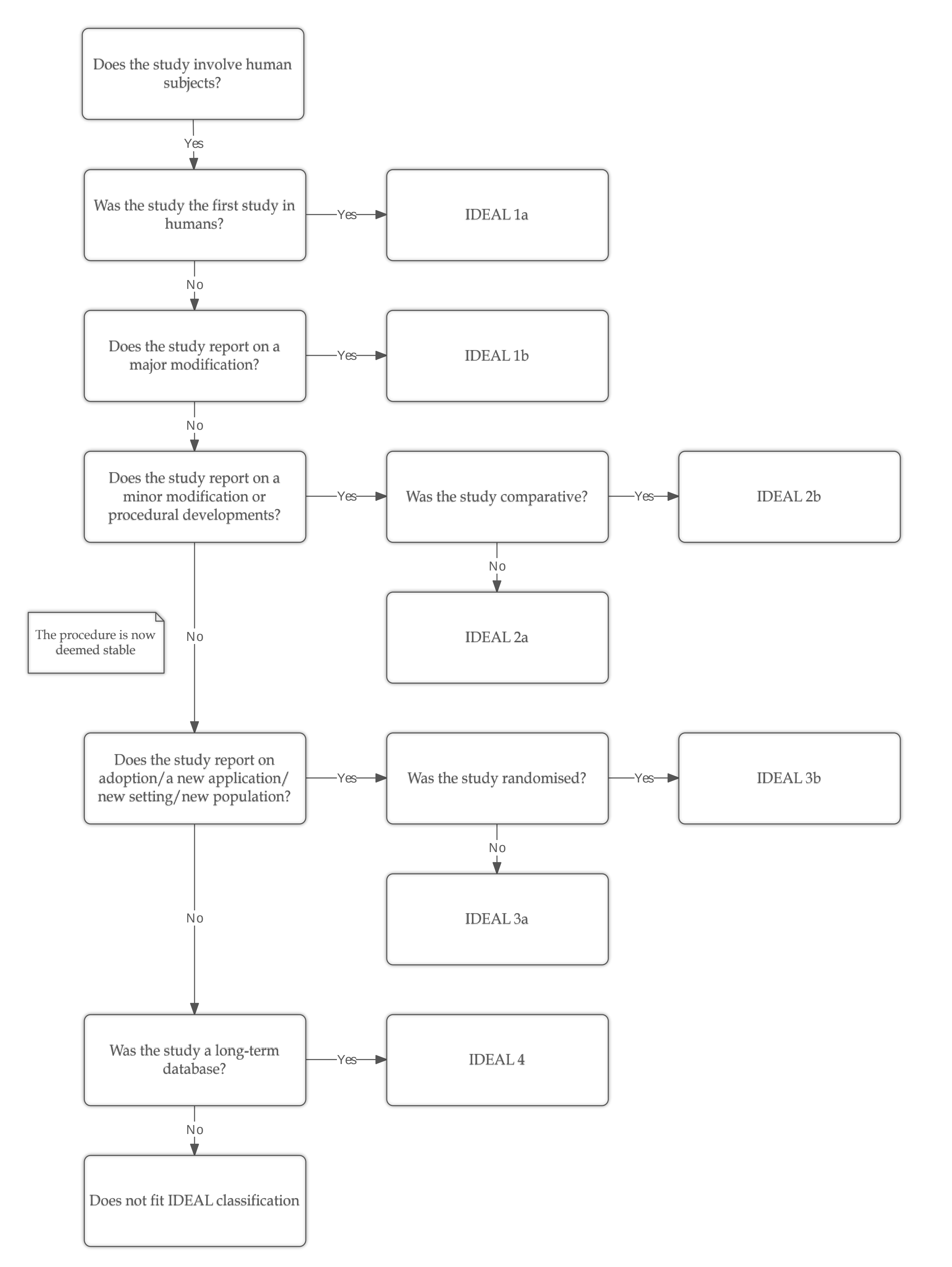

Data extraction checklist Please complete this checklist before moving on to the next paper

Have you:

		YES	NO
For all papers	Completed section A?		
For all papers reporting a pre-clinical study	Completed section B?		
For all papers reporting an animal study	Completed section C?		
For all papers reporting a clinical study	Completed section E?		
	Completed section F?		
	Completed section G?		
	Completed section H?		
	Completed section I?		
	Completed section M?		
	Completed section N?		
	Completed section O?		
	Completed section P?		
	Completed section X?		
	Completed section Y?		
	Completed section Z?		
For papers reporting a comparative clinical study	Completed section J?		
	Completed section K?		
	Completed section L?		
For all papers reporting a clinical study	Completed the relevant critical appraisal pages?		

Did you answer 'UNSURE' to any of the data extraction questions?

If **YES**, put paper forward for discussion with senior author

Tick here ☐

If **NO**, data extraction is complete.

Tick here ☐

THANK YOU. END OF DATA EXTRACTION FORM
