# Supplementary material for: Protocol for the systematic review of the reporting of transoral robotic surgery
Source: BMJ Open. 2018 Jan 23;8(1):e019198. doi: 10.1136/bmjopen-2017-019198 (PMC5786071; doi:10.1136/bmjopen-2017-019198)
Supplement: Author's manuscript [file bmjopen-2017-019198.draft_revisions.pdf]

BMJ Open

BMJ Open is committed to open peer review. As part of this commitment we make the peer review history of every article we publish publicly available.

When an article is published we post the peer reviewers' comments and the authors' responses online. We also post the versions of the paper that were used during peer review. These are the versions that the peer review comments apply to.

The versions of the paper that follow are the versions that were submitted during the peer review process. They are not the versions of record or the final published versions. They should not be cited or distributed as the published version of this manuscript.

BMJ Open is an open access journal and the full, final, typeset and author-corrected version of record of the manuscript is available on our site with no access controls, subscription charges or pay-per-view fees (http://bmjopen.bmj.com).

If you have any questions on BMJ Open's open peer review process please email editorial.bmjopen@bmj.com

BMJ Open

A protocol for the systematic analysis of transoral robotic surgery: a case study of surgical innovation reporting

Journal:	*BMJ Open*
Manuscript ID	bmjopen-2017-019198
Article Type:	Protocol
Date Submitted by the Author:	16-Aug-2017
Complete List of Authors:	Main, Barry; University of Bristol, Population Health Sciences<br>Blencowe, Natalie; University of Bristol, Population Health Sciences<br>Howes, Noah; University of Bristol, Population Health Sciences<br>Cousins, Sian; University of Bristol, Population Health Sciences<br>Avery, Kerry; University of Bristol, Popultation Health Sciences<br>Gormley, Alexander; University of Bristol, Bristol Dental School<br>Radford, Phil; University of Bristol, Bristol Dental School<br>Elliott, Daisy; University of Bristol, Population Health Sciences<br>Byrne, Benjamin; University of Bristol, Population Health Sciences<br>Wilson, Nicholas; University of Bristol, Population Health Sciences<br>Hinchliffe, Robert; University of Bristol, Population Health Sciences<br>Blazeby, Jane; University of Bristol, Population Health Science
**Primary Subject Heading**:	Surgery
Secondary Subject Heading:	Health services research, Research methods, Ear, nose and throat/otolaryngology, Evidence based practice
Keywords:	Head & neck surgery < SURGERY, surgical innovation, robotic surgery

SCHOLARONE™
Manuscripts

For peer review only - http://bmjopen.bmj.com/site/about/guidelines.xhtml

A protocol for the systematic analysis of transoral robotic surgery: a case study of surgical innovation reporting

B G Main[1,2,3], N S Blencowe[1,2], N Howes[2,5] S Cousins[1,2], K N L Avery[1,2], A Gormley[4], P Radford[4], D Elliott[1,2], B Byrne[1,2], N Wilson[1,2], R Hinchliffe[1,2,5], J M Blazeby[1,2,3]

[1]National Institute for Health Research Bristol Biomedical Research Centre, [2] Bristol Centre for Surgical Research, Population Health Sciences, Bristol Medical School, Bristol, UK; [3]University Hospitals Bristol NHS Foundation Trust, Bristol, UK; [4]Bristol Dental School, University of Bristol, Bristol, UK. [5] North Bristol NHS Trust, Bristol, UK.

Correspondence to:

Barry G Main

Bristol Centre for Surgical Research

Population Health Sciences

Bristol Medical School

University of Bristol

Canynge Hall

39 Whatley Road

Bristol

BS8 2PS

Tel: 0044 (0)117 331 3932

Email: b.g.main@bristol.ac.uk

For peer review only

Abstract

Introduction

Transoral robotic surgery (TORS) has been adopted in some parts of the world as an innovative approach to the resection of oropharyngeal tumours. The development, details and outcomes of early-to-later phase evaluation of this technique, and the quality of evidence to support its adoption into practice have hitherto not been summarised. The aim of this review is to identify and summarise the early and later phase studies of, and evidence for, TORS and to understand how early phase studies report intervention development, governance procedures and selection and reporting of outcomes to optimise methods for using the IDEAL (Idea, Development, Exploration, Assessment, Long-term follow-up) framework for surgical innovation that informs evidence-based practice. The protocol has been written in line with the Preferred Reporting Items for Systematic review and Meta-Analysis Protocols (PRISMA-P) checklist.

Methods and analysis

Electronic searches in OVID SP versions of Medline and EMBASE, the Cochrane Central Register of Controlled Trials, and the Cochrane Database of Systematic Reviews, from the start of indexing to the 30th April 2017 will identify studies reporting TORS. At least two independent researchers will identify studies for inclusion. Two researchers will extract data from each paper. Studies will be categorised into IDEAL stages of study design from 'pre-IDEAL' to randomised controlled trials (stage 3). Data will be collected about i) the novel intervention and criteria for modification, ii) governance arrangements and patient information provision, iii) outcome domains selected and reported and iv) quality of study design, conduct and reporting. Descriptive statistics and a narrative synthesis will be presented.

Ethics and dissemination

The results of this systematic review will be presented at relevant conferences. The methods will be used to inform future reviews exploring other novel surgical innovations. The findings will be published in a peer-reviewed journal. This study does not require ethical approval.

For peer review only - http://bmjopen.bmj.com/site/about/guidelines.xhtml

Strengths and weaknesses of this study

- This will be a comprehensive review of trans-oral robotic surgery and will track its innovative evolution since first published description to present day
- Inclusion of all study types will allow identification of good and poor examples of the descriptions of innovative invasive procedures
- The methods described are applicable to reviews of any innovative surgical or other invasive procedure
- Lack of a sensitive literature search strategy may result in large numbers of abstracts to screen at the initial stages
- Exclusion of papers not published in English may mean that important additional findings are missed

For peer review only

Introduction

There is a need to improve outcomes for patients with oropharyngeal cancer. Whilst surgery offers a chance of cure, it carries major risks and there can be long-lasting detrimental effects on function and quality of life.[1] Recent developments in treatment strategies have, therefore, been aimed at organ preservation as well as overall survival.[2] Such developments have included attenuation in the field and dose of radiotherapy delivery, and the introduction of innovative, less destructive surgical approaches including transoral laser and robotic techniques.

Transoral robotic surgery (TORS) is a technique first described in 2005 for the treatment of a benign pharyngeal cyst.[3] It has since been applied to the resection of oropharyngeal cancers and the treatment of obstructive sleep apnoea, was approved by the Food and Drug Administration for these indications in 2009, and subsequently adopted into routine practice in several centres, particularly in the United States.[2] The National Health Service in England do not routinely commission TORS for the treatment of oropharyngeal or laryngeal cancer.[4] The technique involves robotically-assisted excision of a tumour or other tissue by an experienced surgeon who operates a control console in the same room but remote from the patient. Putative benefits include less invasive surgical access, better in situ three-dimensional visualisation of the tumour, scaling of movement, and elimination of hand tremor.[5] Reported disadvantages include lack of tactile feedback and high cost.[6, 7] There appears, however, to be a lack of synthesised data about the effectiveness of TORS. Factors associated with the development, evaluation, and implementation of this innovation have not yet been collated, described or summarised. The extent to which key issues around understanding surgical innovations, including clinical indications for the innovation, which patients are offered the innovation, what information is given to patients to ensure appropriate informed consent in the context of an innovative treatment (and within what governance structure), how the intervention is performed, which modifications are required during development (and what criteria inform decision to modify an intervention), and which outcomes and adverse events are reported in order to document known and potentially unknown consequences of new procedures have been considered also require clarification.

The IDEAL framework (Idea, Development, Exploration, Assessment, Long-term follow-up) was developed in 2009 and proposed a prospective, stepwise means of evaluating innovative

For peer review only

procedures.[8, 9] It aims to help researchers and authors design and report studies of surgical innovation in a transparent way. Widespread adoption of the IDEAL framework by surgical innovators, researchers, and journals has yet to be realised and it is not known if the evolution of technologies like TORS has been reported in accordance with this framework. An in-depth analysis of published literature on TORS will provide a case study of the reporting of a recently-developed surgical intervention which is now offered as part of routine practice.

Overall objective

This study aims to systematically identify and comprehensively summarise and critique studies reporting the introduction, evolution and evaluation of transoral robotic surgery for obstructive sleep apnoea and oropharyngeal cancer.

Specific research questions

- The novel intervention and criteria for modification
  - i) How is the novel intervention reported and criteria for modification described?
  - ii) How were patients selected to receive the intervention? Did these criteria change over time?
  - iii) Were consecutive patients considered for eligibility to be offered the novel intervention? If not, were reasons provided?
  - iv) Were details of patients treated during the same time frame, and who did not receive the novel intervention, reported (including reasons why they were not offered the new treatment, or whether they declined it, and what treatment they did receive)?
  - v) How was surgeon expertise/learning curve reported?
  - vi) Do the studies map onto the IDEAL stages of evaluation of surgical innovation, and is there evidence that the IDEAL stage evolves over time?
  - vii) How are the novel intervention (TORS) and co-interventions and/or comparators defined in the included studies?
  - viii) What is the overall quality of reporting of the included studies as measured using validated tools for randomised and non-randomised studies?

For peer review only

- Governance arrangements
  - ix) How are ethical/governance considerations reported? For example, what descriptions of the process of obtaining the informed consent of patient participants are provided, if any? (note: the study protocols may be consulted in addition to the main report)
  - x) Were patients informed about the innovative nature of the procedure?

- Outcome domains reported
  - xi) Which clinical and patient-reported outcomes, and adverse events, are measured and reported in these studies? How are these outcomes defined and the time points for measurement selected? Did the studies report 'failures' as well as 'successes'?
  - xii) Were outcomes relating to the operator and/or team reported?
  - xiii) Which economic (e.g. cost effectiveness) or other outcomes relating to healthcare resource use (e.g. operating time) were reported?

Methods

Eligibility criteria

Studies will be included in the review if they meet the following inclusion criteria:

Participants – all patients (children and adults) being treated for benign or malignant oropharyngeal disease. In addition to clinical studies, pre-clinical animal and cadaveric studies will be included for review so that the translation from these to first-in-human studies can be mapped.

Intervention – 'transoral robotic surgery' or 'robotically-assisted surgery' or 'robotic surgery for oropharyngeal cancer' or 'robotic surgery for obstructive sleep apnoea' or 'robotic surgery for benign oropharyngeal disease'. Studies will not be limited to a single type, or manufacturer, of robot, or a particular technique.

Comparator(s) – alternative surgical approaches (for example, transoral laser surgery) and/or non-surgical treatments (for example, radiotherapy and/or chemotherapy). It is anticipated that in certain types of early phase studies there will be no comparator group.

Outcome(s) – clinical outcomes, patient-reported outcomes, adverse events, complications and early technical or process outcomes, reported criteria for modifying or withdrawing the intervention, resources use, cost and economic outcomes, and reports of surgeons' experiences of the new procedure (where documented)

For peer review only

Search strategy and study selection

Searches will be undertaken in OVID SP versions of Medline, and EMBASE, the Cochrane Central Register of Controlled Trials and the Cochrane Database of Systematic Reviews. The search strategy will be developed with the assistance of an information specialist. Search terms for transoral robotic surgery, surgery for oropharyngeal cancer, non-surgical treatments of oropharyngeal cancer, surgery for obstructive sleep apnoea and benign oropharyngeal disease will be combined using the Boolean "OR" operator. Pre-clinical (in vitro, animal, and cadaveric studies) will be included only where they have been cited in a clinical study. The search will span from the start of indexing to 1st July 2017. Reference lists of included studies will be searched for additional references. Citations will be collated using Endnote© reference management software. Exclusion criteria will be i) conference reports and abstracts, ii) studies of brain, skull base, lung, oesophageal, thyroid, parathyroid, laryngeal, nasopharyngeal, eye, salivary gland or skin cancers.

Identification and selection of papers

A customised inclusion/exclusion form will be used to screen abstracts and to provide an audit trail. Titles and abstracts will be screened independently by three authors (BM, AG, PR). Any conflicts not resolved by discussion between these authors will be referred to the study team for discussion. The full-text versions of papers retained after title and abstract screening will be downloaded for further assessment of their eligibility for inclusion. The review will be conducted in line with the Preferred Reporting Items for Systematic review and Meta-Analysis (PRISMA) checklist.[10] A PRISMA flow diagram will be produced.

Data extraction and management

Data will be extracted independently by at least two assessors for each paper (BM, JB, NW, AG, PR). A customised data extraction from will be used to collect relevant data from each paper. Data of interest will include:

- The novel intervention and criteria for modification
  - i) Author, date of publication, country of origin of study, journal of publication, and rationale for the study

For peer review only

ii) Details of any funding, including from robot manufacturers, and/or other declared or potential conflicts of interest

iii) Participant demographics (age, sex, tumour details)

iv) Details of the type of centre undertaking the intervention, including any workload data reported. The number and grade of surgeon(s) operating, including their experience with TORS (e.g. training received, evidence of preceptorship)

v) Interventions, co-interventions, comparators and whether each was defined. Any definitions provided in the papers will be recorded verbatim

vi) In-depth details of any descriptions of the component parts of the intervention and any comparator (for example, 'incision', 'access', 'dissection')[11] to explore if any specific components are subject to ongoing innovation

vii) Study classification using a modified IDEAL classification tool. The original IDEAL framework does not come with guidance on how to apply it to the assessment of studies already conducted and published.[8] An adaptation of the IDEAL framework has been designed to permit its application to the retrospective analysis of published studies. It has been tested on a limited set of case studies, but is as yet unpublished.[12] This systematic review will use the new tool to analyse included studies and provide further pilot testing of its validity and reliability.

viii) Details of how the authors discuss and make conclusions related to their findings. For example, any descriptions of the need for further evaluation, the readiness of the intervention for adoption into routine practice, or reasons for stopping the use of the intervention

ix) The overall quality of reporting will be assessed using an appropriate validated critical appraisal tool (e.g. CONSORT for RCTs).[13]

- Governance arrangements

x) Information about governance approvals (ethics committee, IRB, clinical effectiveness committee approval, CE mark approval, Food and Drug Administration (FDA) approval, National Institute for Health and Care Excellence (NICE) approval) and whether these approvals were for the device in general or its use for the treatment of specific clinical indications

xi) Information about how informed consent was obtained from patients, including whether or not they were informed about the innovative nature of the procedure (and if so, how? For example, verbal or written information).

For peer review only

xii) Data about how many patients declined the intervention

- Outcome domains reported

xiii) Names of individual outcomes reported and whether these were specified as primary or secondary outcomes

xiv) Definitions provided (if any) for each of the outcomes

xv) Names of any patient-reported outcomes measures (PROMs),

xvi) The PROs will be classified and the scales used within each will be recorded. Any ad-hoc, study-specific, non-validated PROMs will be documented

xvii) Technical outcomes including issues such as intra-operative events and process outcomes relevant to the evaluation of innovative procedures

xviii) Cost and other economic outcomes

Both randomised and non-randomised studies will be assessed for risk of bias using validated tools. Randomised controlled trials will be assessed using the Cochrane Risk of Bias tool[14] to assess i) the adequacy of sequence generation, ii) allocation concealment, iii) blinding of outcome assessors, iv) completeness of outcome data, v) other sources of bias including selection and funding bias. Non-randomised studies will be assessed using the Risk of Bias in Non-Randomised Studies of Interventions (ROBINS-I) tool[15] for bias due to: i) confounding, ii) selection of participants into the study, iii) classification of interventions, iv) deviation from intended interventions, v) missing data, vi) measurement of outcomes, vii) selection of the reported result.

Data syntheses and statistical analyses

Data will be entered into a custom database. Any discrepancies in data extraction will be resolved by discussion with the entire study group.

The findings will be tabulated and, where appropriate, descriptive statistics performed. A narrative synthesis will summarise the findings of the review by organising data into descriptive themes. The analysis will be longitudinal (for example, does the IDEAL stage evolve over the time span of the review) and cross-sectional (for example, how do studies at a given time point differ?). This review will not aim to make conclusions about the relative effectiveness of TORS over other treatments for

For peer review only

oropharyngeal cancer because the focus is on how innovative surgical procedures are reported in the scientific literature. Therefore, no meta-analyses will be performed.

Dissemination

The systematic review will be published in a peer-reviewed journal and presented at appropriate conferences (for example, methodological and surgical). This protocol will be adapted for the analysis of other innovative surgical and invasive procedures.

Conclusion

This systematic review will provide important information about the quality of studies of robotic surgery for oropharyngeal cancer and benign oropharyngeal disease. It will summarise the reporting of the novel intervention from early phase to any later phase studies published and available for review. It is intended to apply the methods described in this protocol to the analyses of reporting of other novel surgical interventions. The findings will inform further work that will aim to improve studies of innovative surgical interventions by setting standards for the conduct and reporting of both early and later-phase trials.

For peer review only

Contributors

BM, NB and JMB conceived the idea for the TORS review. BM prepared the initial protocol draft with input from AG and PR, and contributed to revision of the manuscript. All authors contributed to the development of the idea, drafting and revision of the manuscript. JMB is the lead of the NIHR Bristol Biomedical Research Centre Surgical Innovation theme and formed the methodological ideas to understand surgical innovation with contributions from NB, RH, BM, SC, DE, BB, KA, NW and NH. All authors gave approval for the manuscript to be submitted.

Funding

This study was supported by the NIHR Biomedical Research Centre at the University Hospitals Bristol NHS Foundation Trust and the University of Bristol. The views expressed are those of the authors and not necessarily those of the UK National Health Service, National Institute for Health Research, or Department of Health. This work was undertaken with the support of the MRC ConDuCT-II (Collaboration and innovation for Difficult and Complex randomised controlled Trials In Invasive procedures) Hub for Trials Methodology Research (MR/K025643/1. JMB is a NIHR Senior Investigator. BM and NB are NIHR Academic Clinical Lecturers, and BB is a NIHR Academic Clinical Fellow.

Competing interests

None declared

Provenance and peer review

Not commissioned; externally peer reviewed

For peer review only

References

1Parsons JT, Mendenhall WM, Stringer SP, Amdur RJ, Hinerman RW, Villaret DB, et al. Squamous cell carcinoma of the oropharynx: surgery, radiation therapy, or both. Cancer. 2002;94(11):2967-80.
2Cracchiolo JR, Roman BR, Kutler DI, Kuhel WI, Cohen MA. Adoption of transoral robotic surgery compared with other surgical modalities for treatment of oropharyngeal squamous cell carcinoma. J Surg Oncol. 2016;114(4):405-11.
3McLeod IK, Melder PC. Da Vinci robot-assisted excision of a vallecular cyst: a case report. Ear Nose Throat J. 2005;84(3):170-2.
4NHS England. Clinical commissioning policy: robotic assisted trans-oral surgery for throat and voice box cancers (Clinical commissioning policy 16008/P). 2016. https://www.england.nhs.uk/commissioning/wp-content/uploads/sites/12/2016/07/16008_FINAL.pdf (Accessed 16th August 2017).
5Hockstein NG, O'Malley BW, Jr., Weinstein GS. Assessment of intraoperative safety in transoral robotic surgery. Laryngoscope. 2006;116(2):165-8.
6Genden EM, O'Malley BW, Jr., Weinstein GS, Stucken CL, Selber JC, Rinaldo A, et al. Transoral robotic surgery: role in the management of upper aerodigestive tract tumors. Head Neck. 2012;34(6):886-93.
7Shah S, Goldenberg D. Robotic surgery for oropharyngeal cancer. Rambam Maimonides Med J. 2014;5(2):e0014.
8McCulloch P, Altman DG, Campbell WB, Flum DR, Glasziou P, Marshall JC, et al. No surgical innovation without evaluation: the IDEAL recommendations. Lancet. 2009;374(9695):1105-12.
9McCulloch P, Cook JA, Altman DG, Heneghan C, Diener MK, Group I. IDEAL framework for surgical innovation 1: the idea and development stages. BMJ. 2013;346:f3012.
10Moher D, Liberati A, Tetzlaff J, Altman DG, Group P. Preferred reporting items for systematic reviews and meta-analyses: the PRISMA statement. BMJ. 2009;339:b2535.
11Blencowe NS, Mills N, Cook JA, Donovan JL, Rogers CA, Whiting P, et al. Standardizing and monitoring the delivery of surgical interventions in randomized clinical trials. Br J Surg. 2016;103(10):1377-84.
12Howes N. Innovation in surgery. The evolution of evidence for a novel surgical procedure. 2017.
13Schulz KF, Altman DG, Moher D, Group C. CONSORT 2010 statement: updated guidelines for reporting parallel group randomised trials. Int J Surg. 2011;9(8):672-7.
14Higgins JP, Altman DG, Gotzsche PC, Juni P, Moher D, Oxman AD, et al. The Cochrane Collaboration's tool for assessing risk of bias in randomised trials. BMJ. 2011;343:d5928.
15Sterne JA, Hernan MA, Reeves BC, Savovic J, Berkman ND, Viswanathan M, et al. ROBINS-I: a tool for assessing risk of bias in non-randomised studies of interventions. BMJ. 2016;355:i4919.

For peer review only

BMJ Open

A protocol for the systematic review of the reporting of transoral robotic surgery

Journal:	*BMJ Open*
Manuscript ID	bmjopen-2017-019198.R1
Article Type:	Protocol
Date Submitted by the Author:	21-Dec-2017
Complete List of Authors:	Main, Barry; University of Bristol, Population Health Sciences<br>Blencowe, Natalie; University of Bristol, Population Health Sciences<br>Howes, Noah; University of Bristol, Population Health Sciences<br>Cousins, Sian; University of Bristol, Population Health Sciences<br>Avery, Kerry; University of Bristol, Popultation Health Sciences<br>Gormley, Alexander; University of Bristol, Bristol Dental School<br>Radford, Phil; University of Bristol, Bristol Dental School<br>Elliott, Daisy; University of Bristol, Population Health Sciences<br>Byrne, Benjamin; University of Bristol, Population Health Sciences<br>Wilson, Nicholas; University of Bristol, Population Health Sciences<br>Hinchliffe, Robert; University of Bristol, Population Health Sciences<br>Blazeby, Jane; University of Bristol, Population Health Science
**Primary Subject Heading**:	Surgery
Secondary Subject Heading:	Health services research, Research methods, Ear, nose and throat/otolaryngology, Evidence based practice
Keywords:	Head & neck surgery < SURGERY, surgical innovation, robotic surgery

SCHOLARONE™
Manuscripts

A protocol for the systematic review of the reporting of transoral robotic surgery

B G Main[1,2,3], N S Blencowe[1,2], N Howes[2,5] S Cousins[1,2], K N L Avery[1,2], A Gormley[4], P Radford[4], D Elliott[1,2], B Byrne[1,2], N Wilson[1,2], R Hinchliffe[1,2,5], J M Blazeby[1,2,3]

[1]National Institute for Health Research Bristol Biomedical Research Centre, [2] Bristol Centre for Surgical Research, Population Health Sciences, Bristol Medical School, Bristol, UK; [3]University Hospitals Bristol NHS Foundation Trust, Bristol, UK; [4]Bristol Dental School, University of Bristol, Bristol, UK. [5] North Bristol NHS Trust, Bristol, UK.

Correspondence to:

Barry G Main

Bristol Centre for Surgical Research

Population Health Sciences

Bristol Medical School

University of Bristol

Canynge Hall

39 Whatley Road

Bristol

BS8 2PS

Tel: 0044 (0)117 331 3932

Email: b.g.main@bristol.ac.uk

For peer review only

Abstract

Introduction

Transoral robotic surgery (TORS) has been adopted in some parts of the world as an innovative approach to the resection of oropharyngeal tumours. The development, details and outcomes of early-to-later phase evaluation of this technique, and the quality of evidence to support its adoption into practice have hitherto not been summarised. The aim of this review is to identify and summarise the early and later phase studies of, and evidence for, TORS and to understand how early phase studies report intervention development, governance procedures and selection and reporting of outcomes to optimise methods for using the IDEAL (Idea, Development, Exploration, Assessment, Long-term follow-up) framework for surgical innovation that informs evidence-based practice. The protocol has been written in line with the Preferred Reporting Items for Systematic review and Meta-Analysis Protocols (PRISMA-P) checklist.

Methods and analysis

Electronic searches in OVID SP versions of Medline and EMBASE, the Cochrane Central Register of Controlled Trials, and the Cochrane Database of Systematic Reviews, from the start of indexing to the 30th April 2017 will identify studies reporting TORS. At least two independent researchers will identify studies for inclusion. Two researchers will extract data from each paper. Studies will be categorised into IDEAL stages of study design from 'pre-IDEAL' to randomised controlled trials (stage 3). Data will be collected about i) the novel intervention and criteria for modification, ii) governance arrangements and patient information provision, iii) outcome domains selected and reported and iv) quality of study design, conduct and reporting. Descriptive statistics and a narrative synthesis will be presented.

Ethics and dissemination

The results of this systematic review will be presented at relevant conferences. The methods will be used to inform future reviews exploring other novel surgical innovations. The findings will be published in a peer-reviewed journal. This study does not require ethical approval.

For peer review only - http://bmjopen.bmj.com/site/about/guidelines.xhtml

Strengths and weaknesses of this study

- This will be a comprehensive review of trans-oral robotic surgery and will track its innovative evolution since first published description to present day
- Inclusion of all study types will allow identification of good and poor examples of the descriptions of innovative invasive procedures
- The methods described are applicable to reviews of any innovative surgical or other invasive procedure
- Lack of a sensitive literature search strategy may result in large numbers of abstracts to screen at the initial stages
- Exclusion of papers not published in English may mean that important additional findings are missed

For peer review only

For peer review only - http://bmjopen.bmj.com/site/about/guidelines.xhtml

Introduction

There is a need to improve outcomes for patients with oropharyngeal cancer. Whilst surgery offers a chance of cure, it carries major risks and there can be long-lasting detrimental effects on function and quality of life.[1] Recent developments in treatment strategies have, therefore, been aimed at organ preservation as well as overall survival.[2] Such developments have included attenuation in the field and dose of radiotherapy delivery, and the introduction of innovative, less destructive surgical approaches including transoral laser and robotic techniques.

Transoral robotic surgery (TORS) is a technique first described in 2005 for the treatment of a benign pharyngeal cyst.[3] It has since been applied to the resection of oropharyngeal cancers and the treatment of obstructive sleep apnoea, was approved by the Food and Drug Administration for these indications in 2009, and subsequently adopted into routine practice in several centres, particularly in the United States.[2] The National Health Service in England do not routinely commission TORS for the treatment of oropharyngeal or laryngeal cancer.[4] The technique involves robotically-assisted excision of a tumour or other tissue by an experienced surgeon who operates a control console in the same room but remote from the patient. Putative benefits include less invasive surgical access, better in situ three-dimensional visualisation of the tumour, scaling of movement, and elimination of hand tremor.[5] Reported disadvantages include lack of tactile feedback and high cost.[6, 7] There appears, however, to be a lack of synthesised data about the effectiveness of TORS. Factors associated with the development, evaluation, and implementation of this innovation have not yet been collated, described or summarised. The extent to which key issues around understanding surgical innovations, including clinical indications for the innovation, which patients are offered the innovation, what information is given to patients to ensure appropriate informed consent in the context of an innovative treatment (and within what governance structure), how the intervention is performed, which modifications are required during development (and what criteria inform decision to modify an intervention), and which outcomes and adverse events are reported in order to document known and potentially unknown consequences of new procedures have been considered also require clarification.

The IDEAL framework (Idea, Development, Exploration, Assessment, Long-term follow-up) was developed in 2009 and proposed a prospective, stepwise means of evaluating innovative

For peer review only - http://bmjopen.bmj.com/site/about/guidelines.xhtml

procedures.[8, 9] It aims to help researchers and authors design and report studies of surgical innovation in a transparent way. Widespread adoption of the IDEAL framework by surgical innovators, researchers, and journals has yet to be realised and it is not known if the evolution of technologies like TORS has been reported in accordance with this framework. An in-depth analysis of published literature on TORS will provide a case study of the reporting of a recently-developed surgical intervention which is now offered as part of routine practice.

Overall objective

This study aims to systematically identify and comprehensively summarise and critique studies reporting the introduction, evolution and evaluation of transoral robotic surgery for obstructive sleep apnoea and oropharyngeal cancer.

Specific research questions

- The novel intervention and criteria for modification
    - i) How is the novel intervention reported and criteria for modification described?
    - ii) How were patients selected to receive the intervention? Did these criteria change over time?
    - iii) Were consecutive patients considered for eligibility to be offered the novel intervention? If not, were reasons provided?
    - iv) Were details of patients treated during the same time frame, and who did not receive the novel intervention, reported (including reasons why they were not offered the new treatment, or whether they declined it, and what treatment they did receive)?
    - v) How was surgeon expertise/learning curve reported?
    - vi) Do the studies map onto the IDEAL stages of evaluation of surgical innovation, and is there evidence that the IDEAL stage evolves over time?
    - vii) How are the novel intervention (TORS) and co-interventions and/or comparators defined in the included studies?
    - viii) What is the overall quality of reporting of the included studies as measured using validated tools for randomised and non-randomised studies?

For peer review only - http://bmjopen.bmj.com/site/about/guidelines.xhtml

- Governance arrangements
    - ix) How are ethical/governance considerations reported? For example, what descriptions of the process of obtaining the informed consent of patient participants are provided, if any? (note: the study protocols may be consulted in addition to the main report)
    - x) Were patients informed about the innovative nature of the procedure?

- Outcome domains reported
    - xi) Which clinical and patient-reported outcomes, and adverse events, are measured and reported in these studies? How are these outcomes defined and the time points for measurement selected? Did the studies report 'failures' as well as 'successes'?
    - xii) Were outcomes relating to the operator and/or team reported?
    - xiii) Which economic (e.g. cost effectiveness) or other outcomes relating to healthcare resource use (e.g. operating time) were reported?

Methods

Eligibility criteria

Studies will be included in the review if they meet the following inclusion criteria:

Participants – all patients (children and adults) being treated for benign or malignant oropharyngeal disease. In addition to clinical studies, pre-clinical animal and cadaveric studies will be included for review so that the translation from these to first-in-human studies can be mapped.

Intervention – 'transoral robotic surgery' or 'robotically-assisted surgery' or 'robotic surgery for oropharyngeal cancer' or 'robotic surgery for obstructive sleep apnoea' or 'robotic surgery for benign oropharyngeal disease'. Studies will not be limited to a single type, or manufacturer, of robot, or a particular technique.

Comparator(s) – alternative surgical approaches (for example, transoral laser surgery) and/or non-surgical treatments (for example, radiotherapy and/or chemotherapy). It is anticipated that in certain types of early phase studies there will be no comparator group.

Outcome(s) – clinical outcomes, patient-reported outcomes, adverse events, complications and early technical or process outcomes, reported criteria for modifying or withdrawing the intervention, resources use, cost and economic outcomes, and reports of surgeons' experiences of the new procedure (where documented)

For peer review only - http://bmjopen.bmj.com/site/about/guidelines.xhtml

For peer review only

Search strategy and study selection

Searches will be undertaken in OVID SP versions of Medline, and EMBASE, PubMed, SCOPUS, Web of Science, the Cochrane Central Register of Controlled Trials and the Cochrane Database of Systematic Reviews. The search strategy will be developed with the assistance of an information specialist. Search terms for transoral robotic surgery, surgery for oropharyngeal cancer, non-surgical treatments of oropharyngeal cancer, surgery for obstructive sleep apnoea and benign oropharyngeal disease (including cysts and papillomas) will be combined using the Boolean "OR" operator (Appendix 1). Pre-clinical (in vitro, animal, and cadaveric studies) will be included only where they have been cited in a clinical study. The search will span from the start of indexing to 1st July 2017. Reference lists of included studies will be searched for additional references. Citations will be collated using Endnote© reference management software. Exclusion criteria will be i) conference reports and abstracts, ii) studies of brain, skull base, lung, oral, hypopharyngeal, parapharyngeal, oesophageal, thyroid, parathyroid, laryngeal, nasopharyngeal, eye, salivary gland or skin cancers.

Identification and selection of papers

A customised inclusion/exclusion form will be used to screen abstracts and to provide an audit trail. Titles and abstracts will be screened independently by three authors (BM, AG, PR). Any conflicts not resolved by discussion between these authors will be referred to the study team for discussion. The full-text versions of papers retained after title and abstract screening will be downloaded for further assessment of their eligibility for inclusion. The review will be conducted in line with the Preferred Reporting Items for Systematic review and Meta-Analysis (PRISMA) checklist.[10] A PRISMA flow diagram will be produced.

Data extraction and management

Data will be extracted independently by at least two assessors for each paper (BM, JB, NW, AG, PR). A customised data extraction from will be used to collect relevant data from each paper (Supplementary file). Data of interest will include:

- The novel intervention and criteria for modification
    - i) Author, date of publication, country of origin of study, journal of publication, and rationale for the study

For peer review only - http://bmjopen.bmj.com/site/about/guidelines.xhtml

ii) Details of any funding, including from robot manufacturers, and/or other declared or potential conflicts of interest

iii) Participant demographics (age, sex, tumour details)

iv) Details of the type of centre undertaking the intervention, including any workload data reported. The number and grade of surgeon(s) operating, including their experience with TORS (e.g. training received, evidence of preceptorship)

v) Interventions, co-interventions, comparators and whether each was defined. Any definitions provided in the papers will be recorded verbatim

vi) In-depth details of any descriptions of the component parts of the intervention and any comparator (for example, 'incision', 'access', 'dissection')[11] to explore if any specific components are subject to ongoing innovation

vii) Study classification using a modified IDEAL classification tool. The original IDEAL framework does not come with guidance on how to apply it to the assessment of studies already conducted and published.[8] An adaptation of the IDEAL framework has been designed to permit its application to the retrospective analysis of published studies. It has been tested on a limited set of case studies, but is as yet unpublished.[12] This systematic review will use the new tool to analyse included studies and provide further pilot testing of its validity and reliability.

viii) Details of how the authors discuss and make conclusions related to their findings. For example, any descriptions of the need for further evaluation, the readiness of the intervention for adoption into routine practice, or reasons for stopping the use of the intervention

ix) The overall quality of reporting will be assessed using an appropriate validated critical appraisal tool (e.g. CONSORT for RCTs).[13]

- Governance arrangements

x) Information about governance approvals (ethics committee, IRB, clinical effectiveness committee approval, CE mark approval, Food and Drug Administration (FDA) approval, National Institute for Health and Care Excellence (NICE) approval) and whether these approvals were for the device in general or its use for the treatment of specific clinical indications

xi) Information about how informed consent was obtained from patients, including whether or not they were informed about the innovative nature of the procedure (and if so, how? For example, verbal or written information).

For peer review only - http://bmjopen.bmj.com/site/about/guidelines.xhtml

xii) Data about how many patients declined the intervention

- Outcome domains reported

xiii) Names of individual outcomes reported and whether these were specified as primary or secondary outcomes

xiv) Definitions provided (if any) for each of the outcomes

xv) Names of any patient-reported outcomes measures (PROMs),

xvi) The PROs will be classified and the scales used within each will be recorded. Any ad-hoc, study-specific, non-validated PROMs will be documented

xvii) Technical outcomes including issues such as intra-operative events and process outcomes relevant to the evaluation of innovative procedures

xviii) Cost and other economic outcomes

Both randomised and non-randomised studies will be assessed for risk of bias using validated tools. Randomised controlled trials will be assessed using the Cochrane Risk of Bias tool[14] to assess i) the adequacy of sequence generation, ii) allocation concealment, iii) blinding of outcome assessors, iv) completeness of outcome data, v) other sources of bias including selection and funding bias. Non-randomised studies will be assessed using the Risk of Bias in Non-Randomised Studies of Interventions (ROBINS-I) tool[15] for bias due to: i) confounding, ii) selection of participants into the study, iii) classification of interventions, iv) deviation from intended interventions, v) missing data, vi) measurement of outcomes, vii) selection of the reported result.

Data syntheses and statistical analyses

Data will be entered into a custom database. Any discrepancies in data extraction will be resolved by discussion with the entire study group.

The findings will be tabulated and, where appropriate, descriptive statistics performed. A narrative synthesis will summarise the findings of the review by organising data into descriptive themes. The analysis will be longitudinal (for example, does the IDEAL stage evolve over the time span of the review) and cross-sectional (for example, how do studies at a given time point differ?). This review will not aim to make conclusions about the relative effectiveness of TORS over other treatments for

For peer review only - http://bmjopen.bmj.com/site/about/guidelines.xhtml

oropharyngeal cancer because the focus is on how innovative surgical procedures are reported in the scientific literature. Therefore, no meta-analyses will be performed.

Dissemination

The systematic review will be published in a peer-reviewed journal and presented at appropriate conferences (for example, methodological and surgical). This protocol will be adapted for the analysis of other innovative surgical and invasive procedures.

Conclusion

This systematic review will provide important information about the quality of studies of robotic surgery for oropharyngeal cancer and benign oropharyngeal disease. It will summarise the reporting of the novel intervention from early phase to any later phase studies published and available for review. It is intended to apply the methods described in this protocol to the analyses of reporting of other novel surgical interventions. The findings will inform further work that will aim to improve studies of innovative surgical interventions by setting standards for the conduct and reporting of both early and later-phase trials.

For peer review only

Contributors

BM, NB and JMB conceived the idea for the TORS review. BM prepared the initial protocol draft with input from AG and PR, and contributed to revision of the manuscript. All authors contributed to the development of the idea, drafting and revision of the manuscript. JMB is the lead of the NIHR Bristol Biomedical Research Centre Surgical Innovation theme and formed the methodological ideas to understand surgical innovation with contributions from NB, RH, BM, SC, DE, BB, KA, NW and NH. All authors gave approval for the manuscript to be submitted. BM is the guarantor.

Funding

This study was supported by the NIHR Biomedical Research Centre at the University Hospitals Bristol NHS Foundation Trust and the University of Bristol. The views expressed are those of the authors and not necessarily those of the UK National Health Service, National Institute for Health Research, or Department of Health. This work was undertaken with the support of the MRC ConDuCT-II (Collaboration and innovation for Difficult and Complex randomised controlled Trials In Invasive procedures) Hub for Trials Methodology Research (MR/K025643/1. JMB is a NIHR Senior Investigator. BM and NB are NIHR Academic Clinical Lecturers, and BB is a NIHR Academic Clinical Fellow.

Competing interests

None declared

Provenance and peer review

Not commissioned; externally peer reviewed

For peer review only

References

1Parsons JT, Mendenhall WM, Stringer SP, Amdur RJ, Hinerman RW, Villaret DB, et al. Squamous cell carcinoma of the oropharynx: surgery, radiation therapy, or both. Cancer. 2002;94(11):2967-80.
2Cracchiolo JR, Roman BR, Kutler DI, Kuhel WI, Cohen MA. Adoption of transoral robotic surgery compared with other surgical modalities for treatment of oropharyngeal squamous cell carcinoma. J Surg Oncol. 2016;114(4):405-11.
3McLeod IK, Melder PC. Da Vinci robot-assisted excision of a vallecular cyst: a case report. Ear Nose Throat J. 2005;84(3):170-2.
4NHS England. Clinical commissioning policy: robotic assisted trans-oral surgery for throat and voice box cancers (Clinical commissioning policy 16008/P). 2016. https://www.england.nhs.uk/commissioning/wp-content/uploads/sites/12/2016/07/16008_FINAL.pdf (Accessed 16th August 2017).
5Hockstein NG, O'Malley BW, Jr., Weinstein GS. Assessment of intraoperative safety in transoral robotic surgery. Laryngoscope. 2006;116(2):165-8.
6Genden EM, O'Malley BW, Jr., Weinstein GS, Stucken CL, Selber JC, Rinaldo A, et al. Transoral robotic surgery: role in the management of upper aerodigestive tract tumors. Head Neck. 2012;34(6):886-93.
7Shah S, Goldenberg D. Robotic surgery for oropharyngeal cancer. Rambam Maimonides Med J. 2014;5(2):e0014.
8McCulloch P, Altman DG, Campbell WB, Flum DR, Glasziou P, Marshall JC, et al. No surgical innovation without evaluation: the IDEAL recommendations. Lancet. 2009;374(9695):1105-12.
9McCulloch P, Cook JA, Altman DG, Heneghan C, Diener MK, Group I. IDEAL framework for surgical innovation 1: the idea and development stages. BMJ. 2013;346:f3012.
10Moher D, Liberati A, Tetzlaff J, Altman DG, Group P. Preferred reporting items for systematic reviews and meta-analyses: the PRISMA statement. BMJ. 2009;339:b2535.
11Blencowe NS, Mills N, Cook JA, Donovan JL, Rogers CA, Whiting P, et al. Standardizing and monitoring the delivery of surgical interventions in randomized clinical trials. Br J Surg. 2016;103(10):1377-84.
12Howes N. Innovation in surgery. The evolution of evidence for a novel surgical procedure. 2017.
13Schulz KF, Altman DG, Moher D, Group C. CONSORT 2010 statement: updated guidelines for reporting parallel group randomised trials. Int J Surg. 2011;9(8):672-7.
14Higgins JP, Altman DG, Gotzsche PC, Juni P, Moher D, Oxman AD, et al. The Cochrane Collaboration's tool for assessing risk of bias in randomised trials. BMJ. 2011;343:d5928.
15Sterne JA, Hernan MA, Reeves BC, Savovic J, Berkman ND, Viswanathan M, et al. ROBINS-I: a tool for assessing risk of bias in non-randomised studies of interventions. BMJ. 2016;355:i4919.

For peer review only

For peer review only

For peer review only - http://bmjopen.bmj.com/site/about/guidelines.xhtml

Appendix 1. Search strategy for OVID version of Medline

1. Robotic Surgical Procedures/

2. ("robotic surgery" or "robotic surgeries" or "robot surgery" or "robotic surgical").mp.

3. 1 or 2

4. (transoral or trans-oral or "trans oral" or tors).mp.

5. exp Oropharyngeal Neoplasms/

6. ((oropharyngeal or oro-pharyngeal or tonsil* or "tongue base") and (cancer$ or neoplasm$ or carcinoma$ or tumo?r$)).mp.

7. 5 or 6

8. 3 and 4 and 7

9. Robotic Surgical Procedures/

10. ("robotic surgery" or "robotic surgeries" or "robot surgery" or "robotic surgical").mp.

11. 9 or 10

12. (transoral or trans-oral or "trans oral" or tors).mp.

13. exp Oropharyngeal Neoplasms/

14. ((oropharyngeal or oro-pharyngeal or tonsil* or "tongue base") and (cancer$ or neoplasm$ or carcinoma$ or tumo?r$)).mp.

15. 13 or 14

16. 11 and 12 and 15

17. Sleep Apnea, Obstructive/su [Surgery]

18. 17 and 3

19. 18 or 16

For peer review only

Systematic analysis of the reporting of surgical innovation: trans oral robotic surgery

Data Extraction Form

Section A: Citation details and funding arrangements. Complete for ALL PAPERS

A1: Unique citation ID: [ ] *(assigned by project lead)*

A2: 1st author: [ ]

A3: Journal: [ ]

A4: 2016 Impact Factor: [_ _._ _ _]

A5: Publication Year:

A6: Volume: [ ]

A7a: Page start: [ ] A7b: Page end: [ ]

A8: Assessor: [ ] (1=BM, 2=AG, 3=PR, 4=JMB, 5=NW, 6=other - add initials here __)

A9: Date of extraction: _ _/_ _/_ _ _ _

A10: Record any statement about conflict of interest (including declarations of no COI) including which author(s) it refers to and the precise nature of the COI here:

____

____

____

A11a: Does the paper include a statement about sponsorship and/or funding? **YES/NO**

A11b: If YES to A11a, was the funding provided by the manufacturer of the robot? **YES/NO**

A11c: If not funded by the robot manufacturer, what was the source of funding or sponsorship? __________

____

A12: Record the corresponding author's email address here: ___________________________________

END OF SECTION A

IF THE PAPER REPORTS A PRE-CLINICAL STUDY – GO TO SECTION B

IF THE PAPER REPORTS A CLINICAL STUDY – GO TO SECTION D

Section B: Preclinical studies (Complete for papers reporting animal and/or cadaveric studies)

(insert free text or select appropriate code to answer each question)

B1: Type of preclinical study ________ (1= animal stud, 2= cadaveric study, 3=combined)

B2: Country of study: _______ (1=UK, 2=North America, 3=EEA, 4=Multiple, 5=Other)

B3: Did the paper include a clear a priori hypothesis and/or present the rationale for the work? **Yes/No**

B3a: If YES, select the most appropriate category for that rationale: ________ (1=first preclinical study of TORS, 2=first preclinical study of a modification of TORS, 3=surgeon training, 4=testing for safety/efficacy/adverse events, 5=to gain FDA/NICE/Other approval, 6=other)

B3b If OTHER, record the statement here verbatim from the paper:

B4: Does the paper include a statement about the surgeons' previous experience of using TORS? **YES/NO**

B4a: If YES, record that statement here, verbatim from the paper:

B4**:** Does the discussion/conclusion present a clear rationale in favour of, or rejection of, progression to clinical studies? **Yes/No**

B6a: If yes, record the statement here verbatim from the paper:

END OF SECTION B

Section C: Critical appraisal of ANIMAL studies. Complete for all pre-clinical in vivo animal studies

For each of the items, indicate whether or not the criteria are fulfilled in the paper

	**Item**	**Y/N/NA**
C1	Does the TITLE accurately describe the content of the paper?	
C2	Does the ABSTRACT provide an accurate summary of the study including the species of animal used?	
C3	Does the BACKGROUND/INTRODUCTION include sufficient scientific information (incl. relevant references) to understand the motivation and context for the study, and explain the approach and rationale?	
C4	Are there clearly described primary and secondary OBJECTIVES, or statement of a specific HYPOTHESIS?	
C5	Is there a clear ETHICAL STATEMENT indicating the nature of the ethics permissions, relevant licenses, and national or institutional guidelines for the care and use of animals?	
C6	Is the STUDY DESIGN (METHODS) clearly described (e.g. number of groups, steps taken to minimise bias,)	
C7	Does the METHODS section clearly describe how the procedure was carried out?	
	C7a: DRUGS – anaesthesia and analgesia	
	C7b: SURGERY – how the procedure was carried out	
	C7c: What TIME of day it was carried out	
	C7d: WHERE it was carried out	
	C7e: Method of EUTHANASIA	
C8	Are there details of the ANIMALS used (species, strain, sex, age, weight). NB might be expressed as mean/median. Record species here: ________________________	
C9	Are there details of how the animals were HOUSED and CARED FOR (animal husbandry)	
C10	SAMPLE SIZE: Is a sample size calculation provided. Is the sample size justified by the authors?	
C11	ALLOCATION: are details and justification provided for how animals were allocated to a given intervention (or control)	
C12	Are the primary and secondary outcomes clearly defined?	
C13	STATISTICAL METHODS: Are these clearly described?	
C14	BASELINE DATA: For each group, are relevant characteristics reported at baseline? (health status, weight etc)	
C15	NUMBERS ANALYSED: Is there clear reporting of the number of animals in each group included in each analysis?	
C16	OUTCOMES: Are the results of each analysis reported?	
C17	ADVERSE EVENTS: Are all adverse events reported in each group?	
C17	MODIFICATIONS: Are any modifications to reduce adverse events/ improve outcomes described and justified?	
C18	DISCUSSION and INTERPRETATION: Does the discussion take into account the study objectives and hypotheses?	
C19	DISCUSSION: Do the authors reflect on the strengths and weaknesses of their work?	
C20	CONCLUSION: Do the authors comments on the generalisability/translation of their findings?	

END OF SECTION C

Section D: Clinical Studies

Section D: Clinical studies: study design and rationale

D1: Type of study:

Use this algorithm to help determine the most appropriate study design for this paper. Indicate the study design by marking the appropriate circle on the diagram. If you are unsure about the study design, please discuss with the senior author

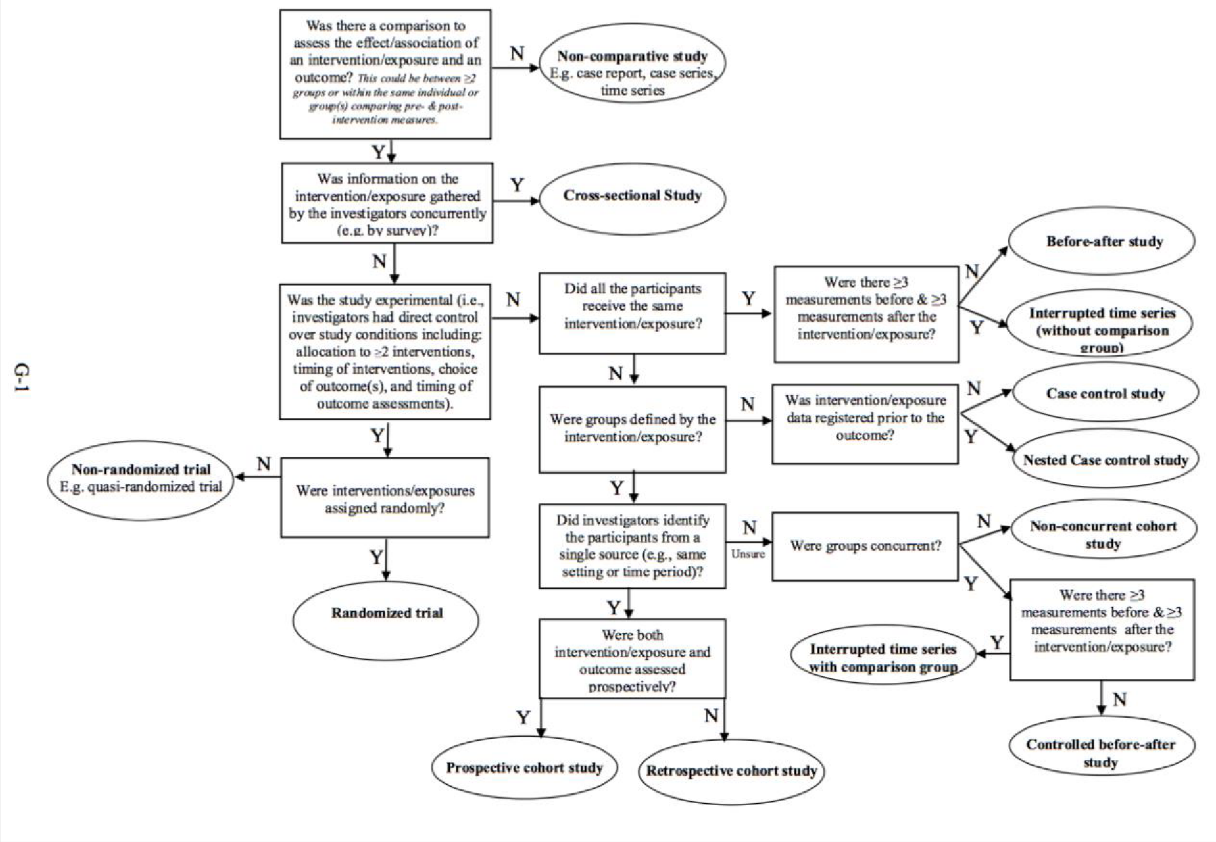

D2: Did the paper include a clear a priori hypothesis and/or present the rationale for the work? **Yes/No**

D2a: If YES, select the most appropriate category for that rationale: ________ (1=first preclinical study of TORS, 2=first preclinical study of a modification of TORS, 3=surgeon training, 4=testing for safety/efficacy/adverse events, 5=to gain FDA/NICE/Other approval, 6=other)

D2b If OTHER, record the statement here verbatim from the paper:

For peer review only - http://bmjopen.bmj.com/site/about/guidelines.xhtml

Section E: Data about the centre and surgeons (Complete for ALL papers reporting a CLINICAL study)

(insert free text or select appropriate code to answer each question)

E1: Country of study: _______ (1=UK, 2=North America, 3=EEA, 4=Multiple, 5=Other)

E2: Centre: _______ (1=single centre, 2=multi centre, NR=not reported)

E3: Number of centres if multiple: ______

E4: Type of centre: _______ (1=general/secondary care, 2=specialist/regional/supraregional/tertiary, 3=not specified)

E5: Is the caseload of the centre(s) reported? (e.g. number of cases normally treated per year) **YES/NO**

E5a: If YES, how many cases? _________

E6: How many surgeons were performing the intervention(s)? _______ (state number or 'NR' if not reported)

E7: Did the authors state any pre-specified criteria for surgeons to be eligible to perform the intervention?

YES/NO *(for example, does the paper state that participating surgeons must have performed it a given number of times?)*

E7a: If YES, what were these criteria and how (if at all) were they justified? (record here, verbatim from paper):

E8: Is there a generic statement about the expertise of those delivering the intervention(s)? **YES/NO** *(for example, does the paper state the number of cases participating surgeons have performed previously?)*

E8a: If YES, please record here verbatim from paper:

E9: If there a statement about how surgeon performance/expertise was monitored? YES/NO

E9a: If YES, please record the statement verbatim from paper here:

END OF SECTION E

For peer review only - http://bmjopen.bmj.com/site/about/guidelines.xhtml

Section F: Data about the patient participants (<u>Complete for ALL papers reporting a clinical study</u>)

(insert free text or select appropriate code to answer each question)

F1: Patients included in the study: ______ (1=adults only, 2= children only, 3=adults and children only, 4=not stated)

F2: Total number of participants: ______

F4: Average age of participants: _______ years (mean/median/mode – circle as appropriate)

F5: Sex of participants: _______ (%=male – calculate from paper if required)

F6: Did the authors include an a priori statement about inclusion and exclusion criteria for patients?

YES/NO

F6a: If yes, record in the table below:

**Inclusion criteria**	**Exclusion criteria**

F7: Were any of the criteria operator dependent (e.g. 'lack of expertise'). **YES/NO**

F7a: If YES, record here:____

F8: Did the authors include a statement about what happened to patients who did not meet the inclusion criteria to receive the intervention (or to participate if it was a comparative study)? **YES/NO**

F8a: If YES, please record the statement here, verbatim from the paper:

F9: Do the authors state that the study included consecutive patients? **YES/NO**

For peer review only - http://bmjopen.bmj.com/site/about/guidelines.xhtml

Section G: Data about governance and ethical factors (<u>Complete for ALL papers reporting a clinical study</u>)

(insert free text or select appropriate code to answer each question)

G1: Was there a statement confirming IRB*/ethics committee approval (**Yes/No**) (* Institutional Review Board)

G2: Was there a statement confirming individual patient consent? (**Yes/No**)

G2a: If yes, record this statement here (verbatim from paper) including any IRB/ethics approval number AND whether this consent was to the innovative procedure OR to the research project OR both:

G3: Was information provided about FDA/NICE/CE marking/clinical effectiveness approval? **YES/NO**

G3a: If YES, record the statement here, including whether that approval was for the device in general (e.g. robot) or for a specific clinical indication (e.g. oropharyngeal cancer):

G4: Was there a statement that patients were <u>specifically</u> informed about the innovative nature of the intervention? (**Yes/No**)

G4a: If yes, record this statement here (verbatim from paper):

G4b: Who gave this information to the patients (e.g. surgeon, nurse, other)? Please record any statement here: ____

G5: Did the paper report any amendment to the IRB/ethics approval AFTER the study had started? Include any statement here (including if the amendment was rejected, why the amendment was sought):

For peer review only - http://bmjopen.bmj.com/site/about/guidelines.xhtml

Section G continued: Data about governance and ethical factors (<u>Complete for ALL papers reporting a clinical study</u>)

G6: Was this study stated to be 'first-in-human' or 'first-in-man'? **YES/NO**

G6a: If YES, was there a statement confirming that the participant(s) were told this? **YES/NO**

G6b: If YES, record that statement here, verbatim from paper:

G7: Was information provided about the number of patients <u>declining</u> the intervention (**Yes/No**)

G7a: If yes, record this information here (verbatim from paper): (NB: this is different from the number of patients found to be ineligible for the intervention as recorded in section F).

G8: Did the authors describe additional sources of information about the intervention(s) provided for patients such as information leaflets, web pages or other sources? **YES/NO**

G8a: If YES, what were these sources:

G8b: If YES, were links or references to these sources included in the paper: **YES/NO**

G8c: If YES, have these sources been obtained? **YES/NO**

<u>If additional sources have been obtained, please retain for further analysis as part of the innovations repository project</u>

Section H: Data about the intervention (TORS) (Complete for ALL papers reporting a clinical study)

(insert free text or select appropriate code to answer each question)

H1: Does the study report at the OUTSET that it was of a modification/refinement/adaptation of TORS? **YES/NO**

H1a: If YES, what was the nature of the modification/refinement/adaptation? ________ (1=use of TORS for a new disease indication, 2=use of TORS in a new patient group, 3=modification of the robot (e.g. addition of an additional piece of equipment), 4=modification of operative technique (e.g. describing a new way for surgeons to operate the robot), 5=other)

H1b: Briefly describe the modification here: ____

____

H1c: If YES, was the rationale/justification for the modification described? **YES/NO**

H1d: If YES, briefly describe that rationale/justification here: ______________________________

____

H2: Does the study report any modifications/refinement/adaptation to TORS that took place AFTER the study had started?

H2a: If YES, what was the nature of the modification/refinement/adaptation? ________ (1=use of TORS for a new disease indication, 2=use of TORS in a new patient group, 3=modification of the robot (e.g. addition of an additional piece of equipment), 4=modification of operative technique (e.g. describing a new way for surgeons to operate the robot), 5=other)

H2b: Briefly describe the modification here: ____

____

H2c: If YES, was the rationale/justification for the modification described? **YES/NO**

H2d: If YES, briefly describe that rationale/justification here: ______________________________

____

H3: If there WAS a modification, is it reported that patients were told about this? **YES/NO**

H4: If YES, record the statement here, verbatim from paper (including any detail about how this information was given – e.g. leaflets):

END OF SECTION H

For peer review only - http://bmjopen.bmj.com/site/about/guidelines.xhtml

Section I: Data about the intervention (contd)) (Complete for ALL papers reporting a clinical study)

This section considers the component parts of the intervention based on Blencowe's typology. Complete the table

	Component	Described in paper? Y/N	If YES, verbatim description from the paper:
I1	Device (TORS) set-up/settings/positioning (not positioning of patient)		
I2	'Pre-incision' (skin preparation, draping, positioning of patient)		
I3	Incision		
I4	Access		
I5	Dissection		
I6	Resection		
I7	Haemostasis		
I8	Insertion of adjuncts (e.g. drains)*		
I9	Closure		

* Do not include tracheostomy here. If tracheostomy is described, please record this in the co-interventions section (section L).

Section J: Data about the <u>comparator</u> interventions (Complete for ALL papers reporting a COMPARATIVE CLINICAL study)

(insert free text or select appropriate code to answer each question) IF THERE IS MORE THAN ONE COMPARATOR (E.G. A THREE-GROUP TRIAL) PLEASE PRINT EXTRA PAGES AS REQUIRED

J1: Which category best describes the comparator: _____ (1= a different transoral surgical technique (e.g. transoral micro laser), 2= other surgery (e.g. lip split and mandibulotomy), 3=radiotherapy, 4= chemotherapy, 5=chemoradiotherapy, 6=other)

J1a: If 'other' please describe here: ____

J2: Does the paper describe an a priori hypothesis/rationale for selecting this comparator? **YES/NO**

J2a: If YES, please record the statement verbatim here:

J3: Does the study report at the OUTSET how patients were selected to be in either the comparator or intervention group(s) **YES/NO** (NB: not applicable if the paper is reporting a RCT)

J3a: If YES, please record the statement here, verbatim from the paper:

END OF SECTION J

Section K: Data about the comparator interventions (contd) (Complete for ALL papers reporting a COMPARATIVE CLINICAL study where the comparator is SURGICAL)

This section considers the component parts of the comparator intervention based on Blencowe's typology. Complete the table if the comparator is surgical. Do not complete if the comparator is non-surgical. PRINT AND COMPLETE MULTIPLE COPIES IF THERE WAS >1 COMPARATOR

	Component	Described in paper? Y/N	If YES, verbatim description from the paper:
K1	Device (e.g. laser or other instrument) set-up/settings/positioning (not positioning of patient)		
K2	'Pre-incision' (skin preparation, draping, positioning of patient)		
K3	Incision		
K4	Access		
K5	Dissection		
K6	Resection		
K7	Haemostasis		
K8	Insertion of adjuncts (e.g. drains)*		
K9	Closure		

* Do not include tracheostomy here. If tracheostomy is described, please record this in the co-interventions section (section L).

Section L: Data about CO-INTERVENTIONS (Complete for ALL papers reporting a CLINICAL study)

Complete the table for each of the co-interventions listed. The INTERVENTION refers to TORS, the comparator to an operative (surgical) comparator group where appropriate. If there was >1 comparator, please print additional copies of the table as required.

	Co-intervention	Description provided in the paper (Y/N)	If YES: Verbatim description of the co-intervention (please include any variance in how the co-intervention was described between intervention and comparator group(s))
L1	Pre-operative imaging		
L2	Pre-operative antibiotics		
L3	Pre-operative analgesia		
L4	Pre-operative fluids		
L5	Pre-operative lines or tubes		
L6	Other pre-op medication		
L7	Thromboprophylaxis		
L8	Other (state)		
L9	Anaesthesia		
L10	Peri-op analgesia		
L11	Peri-op fluids		
L12	Peri-op antibiotics		
L13	Tracheostomy		
L14	Other peri-op lines or tubes		
L15	Peri-op monitoring (pulse oximetry, invasive arterial, temperature)		
L16	Peri-op imaging		
L17	Peri-op thromboprophylaxis		
L18	Other (state)		
L19	Post-op fluids		
L20	Post-op analgesia		
L21	Post-op Antibiotics		
L22	Post-op lines and tubes		
L23	Post-op tracheostomy care		
L24	Intensive care		
L25	Physiotherapy		
L26	Speech and language therapy		
L27	Dietitian therapy		
L28	Other		

For peer review only - http://bmjopen.bmj.com/site/about/guidelines.xhtml

Section L: Data about CO-INTERVENTIONS (contd) (Complete for ALL papers reporting a CLINICAL study)

(insert free text or select appropriate code to answer each question)

L29: Does the study provide a statement about team expertise in delivering the co-intervention(s)? (e.g. anaesthetic team expertise in TORS, operating theatre support staff training or expertise) **YES/NO**

L29a: If YES, please record the statement here verbatim from the paper:

L30: Was there any reported modification to any co-intervention AFTER the study had started? YES/NO

L30a: If YES, describe that modification here:

L31: If this was a comparative study, was the modification in the co-intervention the SAME in both groups? YES/NO

L31A: If NO, how did the modification differ between groups?

L31b: If the co-intervention was different between groups, did the authors provide any rationale for that modification? **YES/NO**

L31c: If YES, please record that statement here verbatim from paper:

For peer review only - http://bmjopen.bmj.com/site/about/guidelines.xhtml

Section M: Outcomes reported (<u>Complete ALL papers reporting a clinical study</u>)

Use this table of outcome domain codes and their descriptors to help complete the next three sections

Domain code	Outcome domain	Definition
Outcome theme: adverse events		
1	Surgical complications	Any short or long term deviation from the normal post-operative course including symptomatic and asymptomatic complications but excluding death
2	Peri-operative technical outcomes	Outcomes recorded directly in the operating theatre, anaesthesia, pre-surgery and immediate recovery before return to the ward/intensive care – e.g. length of procedure, blood loss, or any technical aspects of the procedure
3	Mortality (<30 days)	Outcomes related to short-term survival/death rates and cause of death
4	Mortality (> 30 days)	Outcomes related to long-term survival/death rates and cause of death
Outcome theme: clinical effectiveness		
5	Overall survival	An objective measure of overall survival (at a given time) regardless of cause of death
6	Surrogate survival outcomes	Outcomes relating to survival but which are not overall survival – e.g. disease-free survival, progression-free survival, disease-specific survival
7	Tumour clearance	Outcomes related to achieving tumour clearance (e.g. clear margins)
8	Recurrence of disease	Outcomes related to the recurrence of disease – e.g. cancer or sleep apnoea. Recurrence may be local, regional or distant
9	Treatment pathway outcomes	Outcomes related to the flow of patients through the healthcare system – e.g. length of hospital stay, readmission rates
Outcome theme: physical symptoms, signs and measurements		
10	Haematological or biochemical markers	Outcomes related to the reporting of blood, tissue or biochemical markers – e.g. haemoglobin
11	Physiological measurements	Outcomes related to physiological measurements of function – e.g. sleep studies in treatment of obstructive sleep apnoea
12	Observer assessment of symptoms and function	Signs and symptoms of cancer or sleep apnoea reported to or by clinicians or health care providers directly, and those assessed in clinical practice
Outcome theme: issues relating to quality of life and wellbeing after surgery		
13	Patient-reported outcomes	Qualitative factors, generally assessed by questionnaires, reflecting aspects of a patient's daily life including activities, employment, eating behaviours, psychological health, emotional health, sexual life, sleep function
Outcome theme: health economics		
14	Cost effectiveness	Outcomes related to health economics including cost-effectiveness analyses/modelling, QALYs.
Outcome theme: other/additional/new outcomes		
15	Other	Any outcomes that cannot be categorised into one of the domains listed above

For peer review only - http://bmjopen.bmj.com/site/about/guidelines.xhtml

Section N: Outcomes reported. Please complete for all outcomes reported (you may need to check methods and results sections for details) *Print multiple copies of this page if needed. NB: if any PATIENT-REPORTED OUTOMES, please also complete sections O +/- P*

Outcome name	Domain code	Where reported in paper (1=methods, 2=results, 3=discussion, 4=tables/figures)	Is outcome defined (Y/N)	Verbatim definition	Any citation for a definition (Y/N)	Who ASSESSED the outcome ('NR' if not reported)	Primary or secondary outcome? ('NR' if not reported)

For peer review only - http://bmjopen.bmj.com/site/about/guidelines.xhtml

Section N (contd): Outcomes reported. Please complete for all outcomes reported (you may need to check methods and results sections for details) *Print multiple copies of this page if needed. NB: if any PATIENT-REPORTED OUTOMES, please also complete sections O +/- P*

Outcome name	Domain code	Where reported in paper (1=methods, 2=results, 3=discussion, 4=tables/figures)	Is outcome defined (Y/N)	Verbatim definition	Any citation for a definition (Y/N)	Who ASSESSED the outcome ('NR' if not reported)	Primary or secondary outcome? ('NR' if not reported)

Multiple event – print and insert further copies as required

Section O: Patient reported outcomes – *see Guidance notes for a definition*

O: Complete these questions for any patient reported outcome (one page for each PRO)

O1: Patient reported outcome measure:

i.e. Record verbatim the instrument used to measure the PRO (e.g. QLQ-C30)

O2: If the study was an RCT, was this PRO the 1° outcome? _________ *(1=YES, 2=NO, 3=Not applicable, 4=UNSURE)*

O3: Is this PRO included on the e-provide database (https://eprovide.mapi-trust.org): **YES/NO**

If study is an RCT please complete sections J and K. If not, complete section J only

The following 12 questions are based on the Efficace criteria. See explanatory notes for guidance on completing this section. Tick appropriate box to answer each question.

O3: *A priori* hypothesis stated?

1=DOMAIN	2=DIRECTION	3=BOTH	4=NONE	5=N/A

	1=YES	2=NO	3=N/A
O4: Rationale for instrument reported?			
O5: Psychometric properties reported?			
O6: Cultural validity verified?			
O7: Adequate domains covered?			
O8: Instrument administration reported?			
O9: Baseline compliance reported?			
O10: Timings of assessments documented?			
O11: Missing data documented?			
O12: Clinical significance addressed?			
O13: Presentation of results in general?			

O14: Standard of HRQL reporting:

1=High	2=Low	3=N/A

END OF SECTION O

Multiple event – print and insert further copies as required

Section P: Patient reported outcomes – complete this section ONLY IF RANDOMISED CONTROLLED TRIAL

Section P: CONSORT Patient Reported Outcomes Extension

If the paper is an RCT and includes PROs, please also complete this CONSORT-PRO extension assessment:
(http://jama.jamanetwork.com/article.aspx?articleid=1656259) See explanatory notes for further guidance. Tick appropriate box.

	**CONSORT-PRO extension checklist item**	**1 Yes**	**2 No**	**3 Unsure**
P1	PRO should be identified in abstract as a primary or secondary outcome			
P2	The PRO hypothesis should be stated and relevant domains identified, if applicable			
P3	Evidence of PRO instrument validity should be provided or cited if available, including the person completing the PRO and methods of data collection			
P4	Statistical approaches for dealing with missing data are explicitly stated			
P5	PRO-specific limitations and implications for generalisability and clinical practice are stated			

If 'UNSURE' for any, put paper forward for discussion with senior author.

END OF SECTION P

For peer review only - http://bmjopen.bmj.com/site/about/guidelines.xhtml

Section Q: Critical appraisal: CONSORT checklist for RANDOMISED TRIALS (*tick appropriate box in answer to each checklist item)*

Section/topic		Checklist item	1 YES	2 NO	3 N/A	4 UNSURE
**Title/abstract**	Q1	Identified as RCT in title				
	Q2	Structured abstract of trial design, methods, results, conclusions				
**Introduction**	Q3	Scientific background and explanation of rationale				
	Q4	Specific objectives and hypotheses				
**Methods**	Q5	Description of trial design including allocation ratio				
	Q6	Important changes to methods after trial commencement (e.g. eligibility criteria) explained				
**Eligibility**	Q7	Eligibility criteria for participants described				
**Settings**	Q8	Settings and locations of data collection described				
**Interventions**	Q9	Interventions for each group with sufficient detail to allow replication described				
**Outcomes**	Q10	Pre-specified primary and secondary outcome measures completely defined, including how and when they were assessed				
	Q11	Any changes to trial outcomes after trial commencement described, with reasons				
**Sample Size**	Q12	Explanation of how sample size was determined				
	Q13	If applicable, explanation of any interim analyses and stopping guidelines				
**Randomisation**	Q14	Description of method used to generate random allocation sequence				
	Q15	Description of type of randomisation				
	Q16	Description of mechanism used to implement random allocation sequence (e.g. sequentially numbered containers) including steps taken to conceal the sequence until interventions were assigned				
	Q17	Description of who generated the random allocation sequence, who enrolled participants, and who assigned participants to interventions				
**Blinding**	Q18	Description of any blinding if applicable				
**Statistics**	Q19	Description of statistical methods used to compare groups for primary and secondary outcomes				
		Description used for additional analyses (e.g. subgroup analyses, adjusted analyses)				
**Results**	Q20	Description of participant flow (in each group, number of participants randomly assigned, who received intended treatment, analysed for each primary outcome)				
	Q21	For each group, description of losses and exclusions after randomisation, with reasons				
	Q22	Dates defining the periods of recruitment and follow-up				
	Q23	Description of why the trial ended or was stopped				
**Baseline data**	Q24	Includes a table showing baseline demographics and clinical characteristics for each group				
	Q25	Details of number of participants (denominator) from each group included in each analysis and whether analysis was by original assigned group				
**Outcomes and estimation**	Q26	Results and an estimation of effect size and its precision (e.g. 95% CI) for each primary and secondary outcome for each group				
	Q27	Where applicable, for binary outcomes, inclusion of both absolute and relative effect sizes (*NB only a recommendation rather than requirement in CONSORT*)				
	Q28	Reporting of results of any other analyses performed (e.g. subgroup analyses)				
**Harms**	Q29	Reporting of all important harms or unintended effects in each group				
**Discussion**	Q30	Description of trial limitations, addressing of sources of potential bias, imprecision				
**Generalisability**	Q31	Discussion of generalisability (external validity, applicability) of trial results				
**Interpretation**	Q32	Interpretation that is consistent with results, and that balances benefits and harms and considers other relevant evidence				
**Other information**	Q33	Registration number and name of trial registry included				
	Q34	Details of where to access the full trial protocol, if available				
	Q35	Statement of sources of funding and other support (e.g. supply of drugs) and the role of any funders				

Refer to guidance notes for full explanation of terms

END OF SECTION Q

Section R: Cochrane tool for assessment of risk of bias for <u>RANDOMISED TRIALS</u>

See explanatory notes for guidance on completing, and/or the Cochrane website:

Tick appropriate box to answer each question

(http://ohg.cochrane.org/sites/ohg.cochrane.org/files/uploads/Risk%20of%20bias%20assessment%20tool.pdf)

	**Judgement item**	**1 YES**	**2 NO**	**3 UNSURE**
R1	Was the allocation sequence adequately generated?			
R2	Was the allocation adequately concealed?			
R3	Was knowledge of the allocated intervention adequately prevented during the study? (i.e. was there adequate blinding?)			
R4	Were incomplete outcome data adequately addressed?			
R5	Are reports of the study free of suggestion of selective outcome reporting?			
R6	Was the study apparently free of other problems that could it at a high risk of bias?			
R7	If NO, please specify other sources of bias: *(free text)*			
R8:	What is the overall risk of bias? *(use descriptor and code key to apply overall risk level)* **CODE KEY: 1=LOW, 2=MODERATE, 3=HIGH**	**If any 'UNSURE' discuss with senior author**		

**Risk level**	Description	**Code**
**LOW**	More than 4 'YES' responses	
**MODERATE**	3-4 'YES' responses	
**HIGH**	Fewer than 3 'YES' responses	

R9: Any other comments on bias? *(free text)*

END OF SECTION R

Section S: Critical appraisal: Complete this if paper is a CASE STUDY/CASE REPORT

Select most appropriate response for each statement

	Yes	No	Unclear	Not applicable
S1: Were the patient's demographic characteristics clearly described?				
S2: was the patient's history clearly described and presented as a timeline				
S3: Was the current clinical condition of the patient on presentation clearly described?				
S4: Were diagnostic tests or assessment methods and the results clearly described?				
S5: Was the intervention(s) or treatment procedure(s) clearly described?				
S6: Was the post-intervention clinical condition clearly described?				
S7: Were adverse events (harms) or unanticipated events identified and described?				
S8: Does the case report provide takeaway lessons?				

END OF SECTION S

For peer review only - http://bmjopen.bmj.com/site/about/guidelines.xhtml

Section T: Critical appraisal: Complete this if paper is a CASE SERIES

Select most appropriate response for each statement

	**Yes**	**No**	**Unclear**	**Not applicable**
T1: Were there clear criteria for inclusion in the case series?				
T2: Was the condition measured in a standard, reliable way for all participants included in the case series?				
T3: Were valid methods used for identification of the condition of all participants included in the case series?				
T4: Did the case series have consecutive inclusion of participants?				
T5: Did the case series have complete inclusion of participants?				
T6: Was there clear reporting of the demographics of the participants in the study?				
T7: Was there clear reporting of clinical information of the participants?				
T8: Were the outcomes or follow-up results of cases clearly reported?				
T9: Was there clear reporting of the presenting site(s)/clinic(s) demographic information?				
T10: Was statistical analysis appropriate?				

END OF SECTION T

For peer review only - http://bmjopen.bmj.com/site/about/guidelines.xhtml

Section U: Critical Appraisal: Complete this if paper is a COHORT STUDY

Select most appropriate response for each statement

	Yes	No	Unclear	Not applicable
U1: Were the groups similar and recruited from the same population?				
U2: Were the exposures measured similarly to assign people to both exposed and unexposed groups?				
U3: Was the exposure measured in a valid and reliable way?				
U4: Were the confounding factors identified?				
U5: Were strategies to deal with confounding factors stated?				
U6: Were the groups/participants free of the outcome at the start of the study (or at the moment of exposure?)				
U7: Were the outcomes measured in a valid and reliable way?				
U8: Was the follow up time reported and sufficient to be long enough for outcomes to occur?				
U9: Was follow-up complete, and if not, were the reasons to loss to follow-up described and explored?				
U10: Were strategies to address incomplete follow-up utilised?				
U11: Was appropriate statistical analysis used?				

END OF SECTION U

For peer review only - http://bmjopen.bmj.com/site/about/guidelines.xhtml

Section V: Critical Appraisal: Complete this if paper is a CROSS-SECTIONAL STUDY

Select most appropriate response for each statement

	Yes	No	Unclear	Not applicable
V1: Were the criteria for inclusion in the sample clearly defined?				
V2: Were the study subjects and the setting described in detail?				
V3: Was the exposure measured in a valid and reliable way?				
V4: Were objective, standard criteria used for measurement of the condition?				
V5: were confounding factors identified?				
V6: Were strategies to deal with confounding factors stated?				
V7: Were the outcomes measured in a valid and reliable way?				
V8: Was appropriate statistical analysis used?				

END OF SECTION V

For peer review only - http://bmjopen.bmj.com/site/about/guidelines.xhtml

Section W: Critical Appraisal. Complete this if paper is a <u>NON-RANDOMISED COMPARATIVE STUDY ('QUASI-EXPERIMENTAL)</u>

Select most appropriate response for each statement

	Yes	No	Unclear	Not applicable
W1: Is it clear in the study what is the 'cause' and what is the 'effect' (i.e. there is no confusion about which variable comes first)?				
W2: Were the participants included in any comparisons similar?				
W3: Were the participants included in any comparisons receiving similar treatment/care, other than the exposure or intervention of interest?				
W4: Was there a control group?				
W5: Were there multiple measurements of the outcome both pre- and post- the intervention/exposure?				
W6: Was follow-up complete, and if not, was follow-up adequately reported and strategies to deal with loss to follow-up employed?				
W7: Were the outcomes of participants included in any comparisons measured in the same way?				
W8: Were outcomes measured in a reliable way?				
W9: Were appropriate statistical analyses used?				

END OF SECTION W

For peer review only - http://bmjopen.bmj.com/site/about/guidelines.xhtml

Section X: Additional source(s) of information?

X1: Is any additional source required (e.g. from a study protocol)? **Yes/No**

X1a: Has the additional source of data been found? **Yes/no**

X1b: If yes, what source(s)?

X1c: What additional data did this source(s) add that was not found in the full paper?

X1d: has this additional data been entered at the appropriate place on this form? **YES/NO**

X2: Did this paper cite any references to earlier work? **YES/NO**

X2a: If YES, have these additional papers been obtained? **YES/NO**

X2b: If No, why not? (e.g. unable to obtain full paper) ____________________

Any additional papers obtained should have a unique citation ID assigned and full data extraction completed

Any additional sources (PILS, protocols etc) should be saved as PDF and sent to sian.cousins@bristol.ac.uk **for distribution to the appropriate researchers as part of the INFORM project)**

END OF SECTION X

Section Y: Summing up: Considering the DISCUSSION and CONCLUSION of the paper

Please read and re-read the discussion and/or conclusion *section of the paper before completing this section. Note: some of the information applicable to this section may also be found in the introduction*

X1:Select one of the following statements to most closely reflect the authors' conclusion about TORS from the findings of their study

	**Statement**	**Tick here if most appropriate**
Y1a	The authors recommend further INNOVATION (or development/refinement/adaptation) of TORS is required	
Y1b	The authors recommend further EVALUATION of TORS is required (e.g. they recommend evaluation in an RCT)	
Y1c	The authors recommend ADOPTION of TORS into routine clinical practice	
Y1d	The authors recommend STOPPING the use of TORS in routine clinical practice	
Y1e	The authors make a different conclusion to those above. If so, record their conclusion here:	

The following items relate to implementation and how the authors describe it. Please tick the most appropriate box to respond to each question in the table:

	Tick if applies
**Did the authors consider potential challenges in implementing the intervention more widely?**	
Training others/learning curve/workload or volume limiting patient pool	
Cost	
Competing interventions	
Lack of perceived need for a new intervention	
Resistance to change	
Other. Please state here:	
**Did the authors consider the role of any of the following elements in the generalisation of the intervention to other settings?**	
Specific surgeon(s)	
Specific team(s)	
Perioperative care	
Health care system	
Patient population	
Other. Please state here:	
**Did the authors describe any strategies for promoting engagement and change?**	
Raising profile through publication/presentation at conferences	
Workshops	
Formalised credentialing programme	
Engagement with national specialty/disease groups	
Engagement with national regulatory bodies	
Engagement with patient groups	
Other. Please state here:	

Section Z: Summing up: Categorisation of the study using the IDEAL framework. (Please read the guidance notes to help you complete this section)

Z1: Did the authors state an IDEAL stage? YES/NO. If YES, what was that stage? ________________________

Z2: Use the flow diagram below to classify the study by IDEAL stage. Indicate your selection by circling the appropriate box If it is not possible to classify the study, please state the reason here:____________________

____

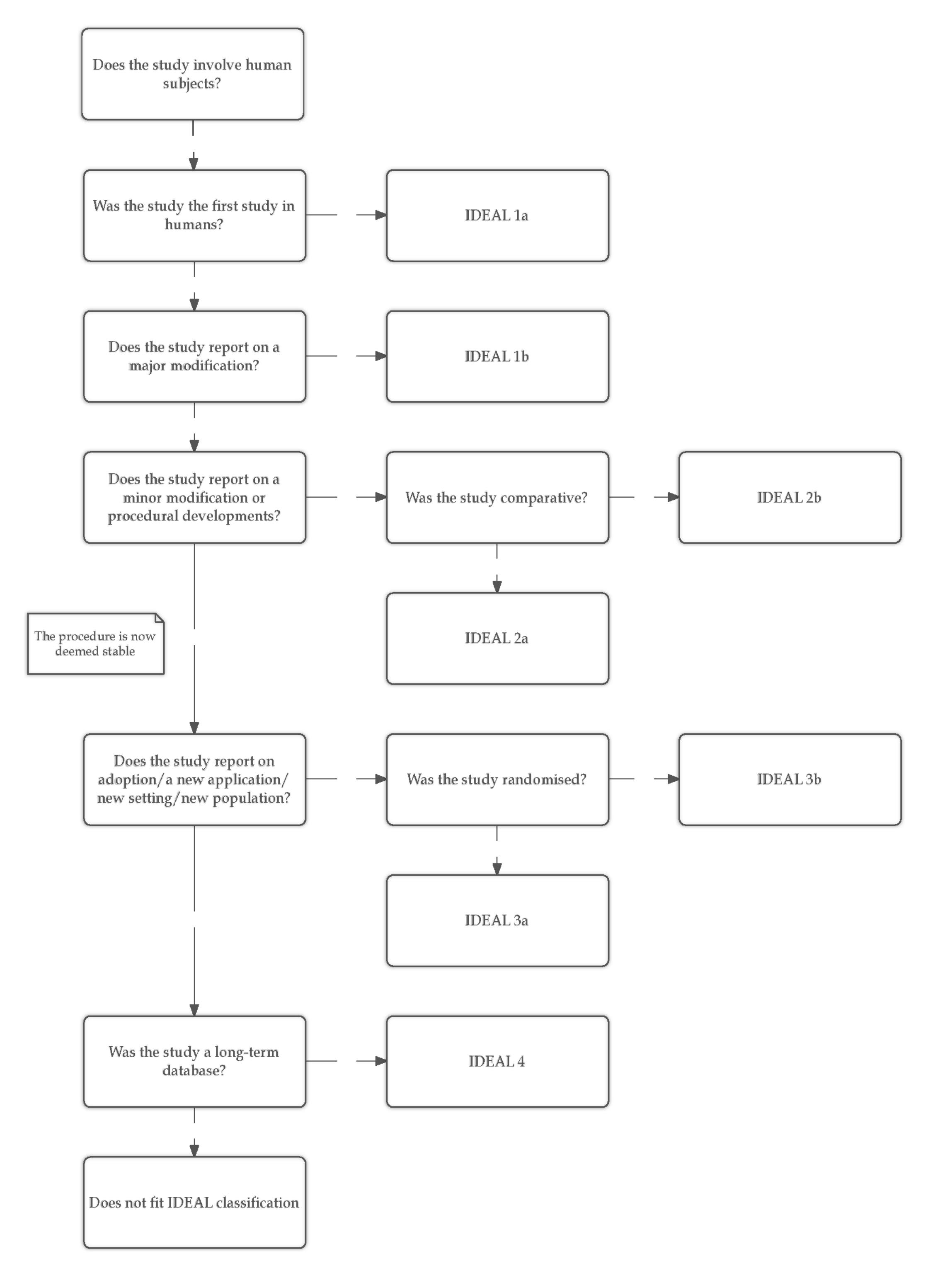

Data extraction checklist Please complete this checklist before moving on to the next paper

Have you:

		YES	NO
For all papers	Completed section A?		
For all papers reporting a pre-clinical study	Completed section B?		
For all papers reporting an animal study	Completed section C?		
For all papers reporting a clinical study	Completed section E?		
	Completed section F?		
	Completed section G?		
	Completed section H?		
	Completed section I?		
	Completed section M?		
	Completed section N?		
	Completed section O?		
	Completed section P?		
	Completed section X?		
	Completed section Y?		
	Completed section Z?		
For papers reporting a comparative clinical study	Completed section J?		
	Completed section K?		
	Completed section L?		
For all papers reporting a clinical study	Completed the relevant critical appraisal pages?		

Did you answer 'UNSURE' to any of the data extraction questions?

If **YES**, put paper forward for discussion with senior author

Tick here ☐

If **NO**, data extraction is complete.

Tick here ☐

THANK YOU. END OF DATA EXTRACTION FORM

For peer review only - http://bmjopen.bmj.com/site/about/guidelines.xhtml

PRISMA-P (Preferred Reporting Items for Systematic review and Meta-Analysis Protocols) 2015 checklist: recommended items to address in a systematic review protocol*

Section and topic	Item No	Checklist item
**ADMINISTRATIVE INFORMATION**		
Title:		
Identification	1a	Identify the report as a protocol of a systematic review Title, page 1
Update	1b	If the protocol is for an update of a previous systematic review, identify as such N/A
Registration	2	If registered, provide the name of the registry (such as PROSPERO) and registration number N/A
Authors:		
Contact	3a	Provide name, institutional affiliation, e-mail address of all protocol authors; provide physical mailing address of corresponding author Page 1
Contributions	3b	Describe contributions of protocol authors and identify the guarantor of the review p11
Amendments	4	If the protocol represents an amendment of a previously completed or published protocol, identify as such and list changes; otherwise, state plan for documenting important protocol amendments N/A
Support:		
Sources	5a	Indicate sources of financial or other support for the review p11
Sponsor	5b	Provide name for the review funder and/or sponsor p11
Role of sponsor or funder	5c	Describe roles of funder(s), sponsor(s), and/or institution(s), if any, in developing the protocol p11 (disclaimer)
**INTRODUCTION**		
Rationale	6	Describe the rationale for the review in the context of what is already known pp 4-5
Objectives	7	Provide an explicit statement of the question(s) the review will address with reference to participants, interventions, comparators, and outcomes (PICO) pp 5-6
**METHODS**		
Eligibility criteria	8	Specify the study characteristics (such as PICO, study design, setting, time frame) and report characteristics (such as years considered, language, publication status) to be used as criteria for eligibility for the review pp 6-7
Information sources	9	Describe all intended information sources (such as electronic databases, contact with study authors, trial registers or other grey literature sources) with planned dates of coverage p7
Search strategy	10	Present draft of search strategy to be used for at least one electronic database, including planned limits, such that it could be repeated added to p13
Study records:		
Data management	11a	Describe the mechanism(s) that will be used to manage records and data throughout the review pp7-8

For peer review only

Selection process	11b	State the process that will be used for selecting studies (such as two independent reviewers) through each phase of the review (that is, screening, eligibility and inclusion in meta-analysis) p7
Data collection process	11c	Describe planned method of extracting data from reports (such as piloting forms, done independently, in duplicate), any processes for obtaining and confirming data from investigators p7
Data items	12	List and define all variables for which data will be sought (such as PICO items, funding sources), any pre-planned data assumptions and simplifications pp7-9
Outcomes and prioritization	13	List and define all outcomes for which data will be sought, including prioritization of main and additional outcomes, with rationale pp7-9
Risk of bias in individual studies	14	Describe anticipated methods for assessing risk of bias of individual studies, including whether this will be done at the outcome or study level, or both; state how this information will be used in data synthesis p9
Data synthesis	15a	Describe criteria under which study data will be quantitatively synthesised N/A
	15b	If data are appropriate for quantitative synthesis, describe planned summary measures, methods of handling data and methods of combining data from studies, including any planned exploration of consistency (such as I^2, Kendall's τ)N/A
	15c	Describe any proposed additional analyses (such as sensitivity or subgroup analyses, meta-regression)N/A
	15d	If quantitative synthesis is not appropriate, describe the type of summary planned p9
Meta-bias(es)	16	Specify any planned assessment of meta-bias(es) (such as publication bias across studies, selective reporting within studies) none planned
Confidence in cumulative evidence	17	Describe how the strength of the body of evidence will be assessed (such as GRADE) a qualitative summary will be prepared. This review does not seek to make conclusions about relative effectiveness that would influence policy

*** It is strongly recommended that this checklist be read in conjunction with the PRISMA-P Explanation and Elaboration (cite when available) for important clarification on the items. Amendments to a review protocol should be tracked and dated.** **The copyright for PRISMA-P (including checklist) is held by the PRISMA-P Group and is distributed under a Creative Commons Attribution Licence 4.0.**

From: Shamseer L, Moher D, Clarke M, Ghersi D, Liberati A, Petticrew M, Shekelle P, Stewart L, PRISMA-P Group. Preferred reporting items for systematic review and meta-analysis protocols (PRISMA-P) 2015: elaboration and explanation. BMJ. 2015 Jan 2;349(jan02 1):g7647.

For peer review only
